# Metabolomics analysis: Finding out metabolic building blocks

**DOI:** 10.1371/journal.pone.0177031

**Published:** 2017-05-11

**Authors:** Ricardo Alberich, José A. Castro, Mercè Llabrés, Pere Palmer-Rodríguez

**Affiliations:** 1 Department of Mathematics and Computer Science, University of the Balearic Islands, Palma, Balearic Islands, Spain; 2 Department of Biology, University of the Balearic Islands, Palma, Balearic Islands, Spain; Friedrich-Alexander-Universitat Erlangen-Nurnberg, GERMANY

## Abstract

In this paper we propose a new methodology for the analysis of metabolic networks. We use the notion of strongly connected components of a graph, called in this context metabolic building blocks. Every strongly connected component is contracted to a single node in such a way that the resulting graph is a directed acyclic graph, called a metabolic DAG, with a considerably reduced number of nodes. The property of being a directed acyclic graph brings out a background graph topology that reveals the connectivity of the metabolic network, as well as bridges, isolated nodes and cut nodes. Altogether, it becomes a key information for the discovery of functional metabolic relations. Our methodology has been applied to the glycolysis and the purine metabolic pathways for all organisms in the KEGG database, although it is general enough to work on any database. As expected, using the metabolic DAGs formalism, a considerable reduction on the size of the metabolic networks has been obtained, specially in the case of the purine pathway due to its relative larger size. As a proof of concept, from the information captured by a metabolic DAG and its corresponding metabolic building blocks, we obtain the core of the glycolysis pathway and the core of the purine metabolism pathway and detect some essential metabolic building blocks that reveal the key reactions in both pathways. Finally, the application of our methodology to the glycolysis pathway and the purine metabolism pathway reproduce the tree of life for the whole set of the organisms represented in the KEGG database which supports the utility of this research.

## Introduction

Metabolism is the chemical system that generates the essential components for life. All living organisms possess an intricate network of metabolic routes for the biosynthesis of amino acids, nucleic acids, lipids and carbohydrates and for the catabolism of different compounds driving cellular processes. Traditionally, metabolism has been divided into metabolic pathways: subsystems of metabolism dealing with specific functions. However, it has become increasingly clear that metabolism operates as a highly integrated network [1].

From this general perspective, co-analysis of phylogeny and metabolic networks can provide valuable insight in explaining the appearance and development of complex networks of interacting proteins and chemical molecules [2, 3], or yielding a valuable information on the metabolism generated by a set of organisms, as for instance the recent results presented in [4] for the gut microbiome. As metabolic evolution along time goes, several theories have been proposed to explain the evolution of these networks (see [5] for a review). Some current research supports the so-called *patchwork evolution model* [6], while others support the so-called *panspermia theory* [7]. Therefore, it is still unclear whether other biological mechanisms played a significant role in the emergence of metabolic networks and the question, “does metabolomics meet genomics?” is still open.

Over the last ten years metabolic pathways have been the subject of a great deal of research, conducted primarily through two kinds of studies, focusing either on the analysis of single pathways [8–10], or on the comparative analysis of a set of pathways [11]. The studies that analyze and compare metabolic pathways of different species can provide interesting information on their evolution and may help to understand metabolic functions, which are important in studying diseases and identifying pharmacological targets. In the literature many techniques have been proposed for comparing metabolic pathways of different organisms [12, 13]. Each approach chooses a representation of metabolic pathways that models the information of interest, proposes a similarity or a distance measure and possibly supplies a tool for performing the comparison. The automation of the whole process is enabled by the knowledge stored in metabolic databases such as KEGG [14], BioModels [15] or MetaCyc [16]. However, dealing with the entire metabolism of an organism, or a set of organisms, increases too much the size of the networks to analyze. This fact makes it necessary to redefine the representation of these huge metabolic networks such that, on one hand it models the information of interest but, on the other hand, it reduces the size of the network. With this idea in mind, we conceived the present research.

Since we are interested in a topological analysis of metabolic networks, we focus on a network based approach instead of other approaches like kinetic modeling, hybrid modeling or constraint based modeling. See [17] for a good review on comparing methods for metabolic pathway analysis. The three basic methods used in the network based approach under a structural and stoichiometric modeling are hypergraph based, elementary flux mode analysis and extreme pathway analysis [18–20]. However, due to the fact that in elementary flux mode analysis and extreme pathway analysis computing the elementary modes and extreme pathways is an NP-hard computational problem, we decided to consider a new methodology based on graph representation instead of a stoichiometric modeling to study the robustness, modularity and connectivity of a metabolic network in polynomial time. Thus, the reason to model metabolic networks as directed graphs is twofold. Firstly, directed graphs are a very simple and well studied formalism able to model the topological information of the network. Secondly, we can consider the well known notion of strongly connected components in directed graphs, which are computed in polynomial time, to reduce appropriately the metabolic network in order to study its network topology.

Hence, we propose a methodology for the analysis of metabolic networks that aims at providing a good balance between the information of interest that must be kept and a considerably reduction of the size of the network to facilitate its analysis and visualization. In this paper we introduce a new approach to metabolic networks modeling based on classical notions of graph theory, which applied to metabolic networks have proved to be successful. Namely, we used the notion of strongly connected components, which in this context we call *metabolic building blocks*. When every metabolic building block in the initial directed graph is contracted to a single vertex, the resulting graph is a directed acyclic graph, called a *metabolic DAG*, whose number of nodes is considerably reduced. Moreover, the property of being a directed acyclic graph brings out a background graph topology that reveals the connectivity of the network as well as bridges, isolated nodes and cut nodes, which become key information for the discovery of functional metabolic relations. The results of the preliminary tests we have carried on, reported in this paper, shows that our methodology fulfills the requirements of network size reduction while preserving information of interest.

## Materials and methods

This section describes the methodology proposed for the analysis of metabolic networks. We represent metabolic pathways and metabolic networks as directed graphs, called reaction graphs. The analysis of every network derives from the study of topological properties of the associated reaction graph. We introduce a suitable graph reduction, called metabolic DAG, which captures the network connectivity and reveals a set of key reactions as well as a modularization of the network. Furthermore, the metabolic networks analysis and comparison is easily obtained from the reaction graph reduction.

### Metabolic networks as reaction graphs

A metabolic network is the complete set of metabolic and physical processes that determine the physiological and biochemical properties of a cell. As such, these networks comprise the chemical reactions of metabolism, the metabolic pathways, as well as the regulatory interactions that guide these reactions. More precisely, a chemical reaction is a process that leads to the transformation of one set of chemical substances or metabolites called substrate, to another called product. Chemical reactions are catalyzed by enzymes that accelerate their rate. Thus, in this paper we denote a chemical reaction by *R*_*i*_ = (*I*_*i*_, *E*_*i*_, *O*_*i*_), *I*_*i*_ being its substrate, *E*_*i*_ the enzyme that catalyzes the reaction and *O*_*i*_ its product.

A *directed graph* is an ordered pair *G* = (*V*, *E*) where *V* is a set of nodes and *E* ⊆ *V* × *V* is a set of arcs. There is an arc from a node *u* ∈ *V* to a node *v* ∈ *V* if, and only if, (*u*, *v*) ∈ *E*. In this work, we model a metabolic network as a directed graph *G*_*R*_ = (*R*, *E*) whose set of nodes is the set *R* of chemical reactions present in the metabolic network, and its set of arcs *E* is defined as follows: there is an arc from *R*_*i*_ = (*I*_*i*_, *E*_*i*_, *O*_*i*_) to *R*_*j*_ = (*I*_*j*_, *E*_*j*_, *O*_*j*_) if, and only if, there exists one metabolite *c* such that *c* ∈ *O*_*i*_ ∩ *I*_*j*_. That is, there is an arc from *R*_*i*_ to *R*_*j*_ if, and only if, at least one metabolite in the product of *R*_*i*_ is in the substrate of *R*_*j*_. Notice that, metabolites are not represented in our modeling, but we take them into account to define the arcs. Most of the reactions in metabolic networks are reversible. A reversible reaction can occur in two directions, from the reactants to the products (forward reaction) or vice versa (backward reaction). The direction depends on the kind of reaction, on the concentration of the metabolites, and on conditions such as temperature and pressure. In this work, we model reversible reactions by two different nodes, one for the forward reaction and the other for the backward reaction. The directed graph *G*_*R*_, modeling a metabolic network, is called a *reaction graph*.

As we have already explained in the introduction section, reaction graphs may be huge. Indeed, with only one metabolic pathway we can get more than a hundred of nodes as it is the case of the purine metabolism pathway in *Homo sapiens*, available at the KEGG database [21], whose reaction graph has 141 nodes and 527 arcs. This amount of nodes and arcs in the reaction graph hinders the visualization of the pathway topology. Thus, it is convenient to suitably reduce the number of nodes in order to visualize clearly the network.

### Metabolic DAGs

As stated above, metabolic networks use to involve thousands of reactions. Therefore, the corresponding reaction graphs have thousands of nodes which difficult their analysis and study. In order to overcome this problem, we introduce metabolic DAGs, which consist on a condensation of the reaction graph that preserves the relations between reactions.

A *directed acyclic graph*, a DAG, is a directed graph with no directed cycles. In a directed graph, *G*, two nodes *u*,*v* are said to be biconnected if there is a path in each direction between them. A *strongly connected component* of a directed graph *G* is a subgraph such that every pair of nodes in it are biconnected, and it is maximal under inclusion with this property [22]. Since biconnectivity is an equivalence relation, the collection of strongly connected components forms a partition of the set of nodes of *G*. If each strongly connected component is contracted to a single vertex, the resulting quotient graph is a DAG, the *condensation* of G. Notice that there is an arc from a node *s*_*i*_ to a node *s*_*j*_ in the condensation of a directed graph *G* if, and only if, there is an arc in *G* from a node *u* ∈ *s*_*i*_ to a node *v* ∈ *s*_*j*_.

Thus, for every reaction graph *G*_*R*_, we can consider its collection of strongly connected components and compute its condensation, which will be a DAG. We call *metabolic building blocks* (MBBs for short) the strongly connected components in the reaction graph *G*_*R*_, and we call metabolic DAG (m-DAG for short) the condensation of *G*_*R*_, which is a DAG whose nodes are the MBBs of *G*_*R*_.

Intuitively, if we consider a reaction graph *G*_*R*_ and its condensation, all the nodes in a MBB are the reactions that are biconnected among them in the reaction graph, that is, such that there is a path back and forward between them. In this sense, MBBs can be thought as robust subgraphs in the reaction graph. Moreover, the reactions that are not biconnected to any other reaction, which become a MBB by themselves in the m-DAG, may be necessary for the connectivity of the network. In this sense, the condensation of *G*_*R*_ provides a modularity of the reaction graph that keeps the information of robustness and connectivity of the metabolic network.

**Fig 1:**
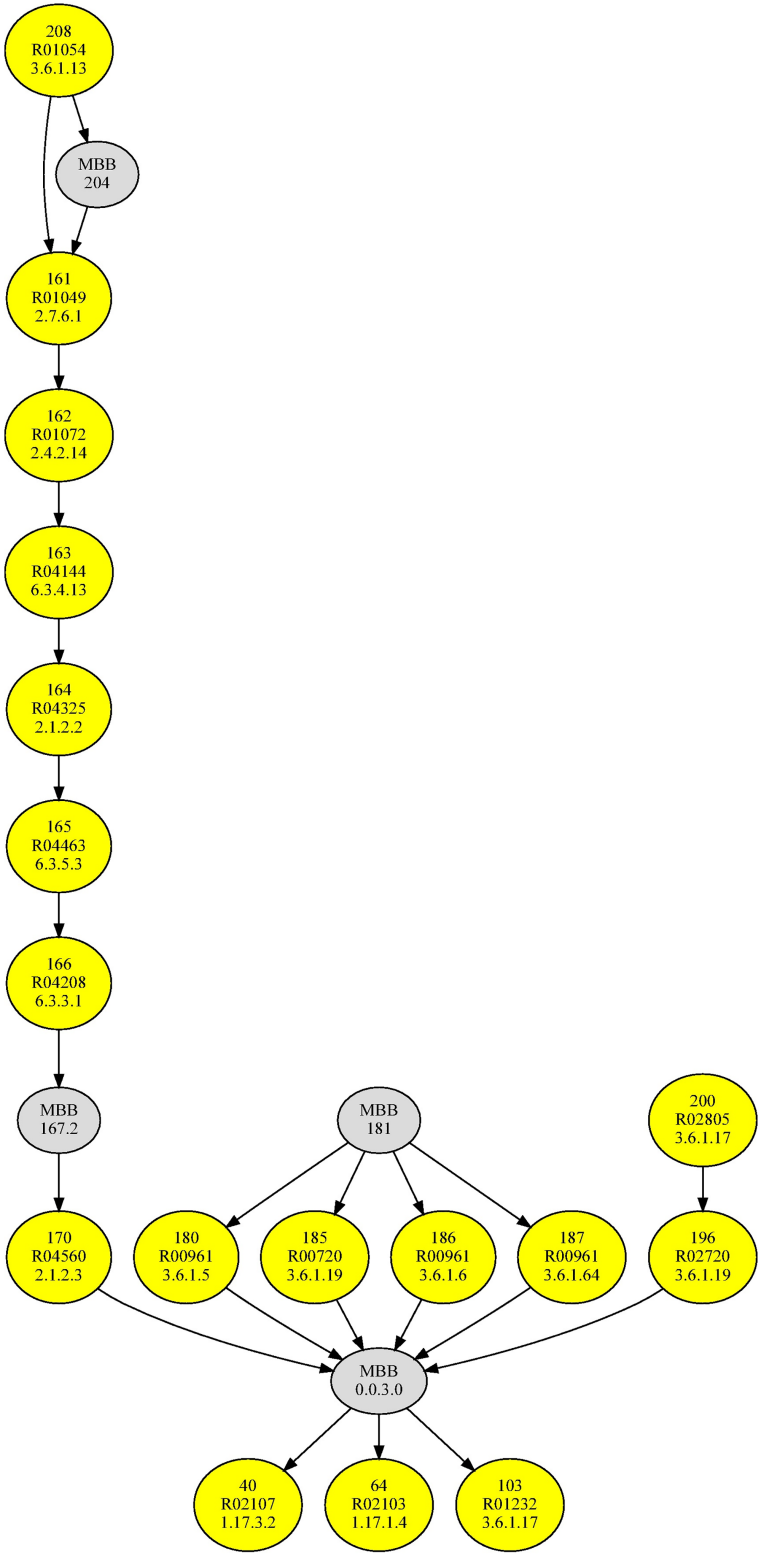
The m-DAG of the purine metabolism pathway in *Homo sapiens*. The nodes in this graph are the MBBs in the reaction graph of the purine metabolism pathway in *Homo sapiens*. Yellow nodes denote MBBs with only one reaction, while grey nodes denote MBBs with more than one reaction.

Fig 1 shows the m-DAG corresponding to the condensation of the reaction graph of the purine metabolism pathway in *Homo sapiens* (http://www.genome.jp/kegg-bin/show_pathway?hsa00230 More specifically, the nodes in the m-DAG are the MBBs in the reaction graph. As we can see in the picture, this m-DAG has only 21 nodes. Only four of them, denoted by grey nodes, are MBBs with more than one reaction, and the remaining, denoted by yellow nodes, are MBBs with only one reaction. Now, we clearly see in the m-DAG that the purine metabolism pathway has a linear path, the chain of yellow nodes in the picture, playing a key role in the pathway connectivity, since the removal of one of this yellow nodes renders the pathway disconnected. That is, they are cut nodes in the m-DAG. Furthermore, every MBB, which is now a smaller directed graph, can be considered as a metabolic network itself and can be analyzed using other well established approaches like elementary flux modes or extreme pathways. Therefore, we claim that with our methodology we obtain a modularization of the reaction graph, where each module is a metabolic building block and the topology of the m-DAG provides with useful information on the topology of the reaction graph. The advantages of considering m-DAGs are the following

1. It reduces considerably the number of nodes.
2. It shows the reaction graph topology and metabolic linear paths of the network.
3. It shows the cut nodes in the metabolic network.

We call *essential reactions* those reactions in *G*_*R*_ that are cut nodes. Then, in terms of m-DAGs, the essential reactions are the MBBs with only one reaction such that they are cut nodes, that is, a node whose removal renders the m-DAG disconnected. Essential reactions can be easily observed in the m-DAG since the number of nodes in this graph is considerably reduced. Indeed, a simple look at the m-DAG in Fig 1 shows that there are eight essential reactions, six of them form the linear path in the m-DAG, MBB 170 connects the linear path with MBB 0.0.3.0, while MBB 196 connects MBB 200 (an entrance to the pathway) with MBB 0.0.3.0. All these reactions are needed to keep the connectivity of the pathway. In addition, the four MBBs with more than one reaction, labelled by 0.0.3.0, 181, 167.2 and 204 have 109, 3, 8 and 4 reactions respectively. Tables in S2 and S6 Tables in the supporting information files show for every organism all the information of these MBBs. We can observe that MBB 0.0.3.0 is considerably bigger than the others, and also that it contains those reactions whose product are either Adenine or Guanine.

In order to better understand the correspondence between a reaction graph and its m-DAG, we also considered as a running example the glycolysis pathway. We decided to test how our methodology performs with the glycolysis pathway for several reasons. First of all, it is not a huge pathway, so it is easy to visualize the relation between the reaction graph and its corresponding m-DAG. Second, this is probably one of the most studied metabolic pathways, so it is available in almost all organisms in the KEGG database. And finally, it is an ancient and conserved pathway among almost all species and we believe that it would be interesting to compare the corresponding m-DAG for all species in the KEGG database.

**Fig 2:**
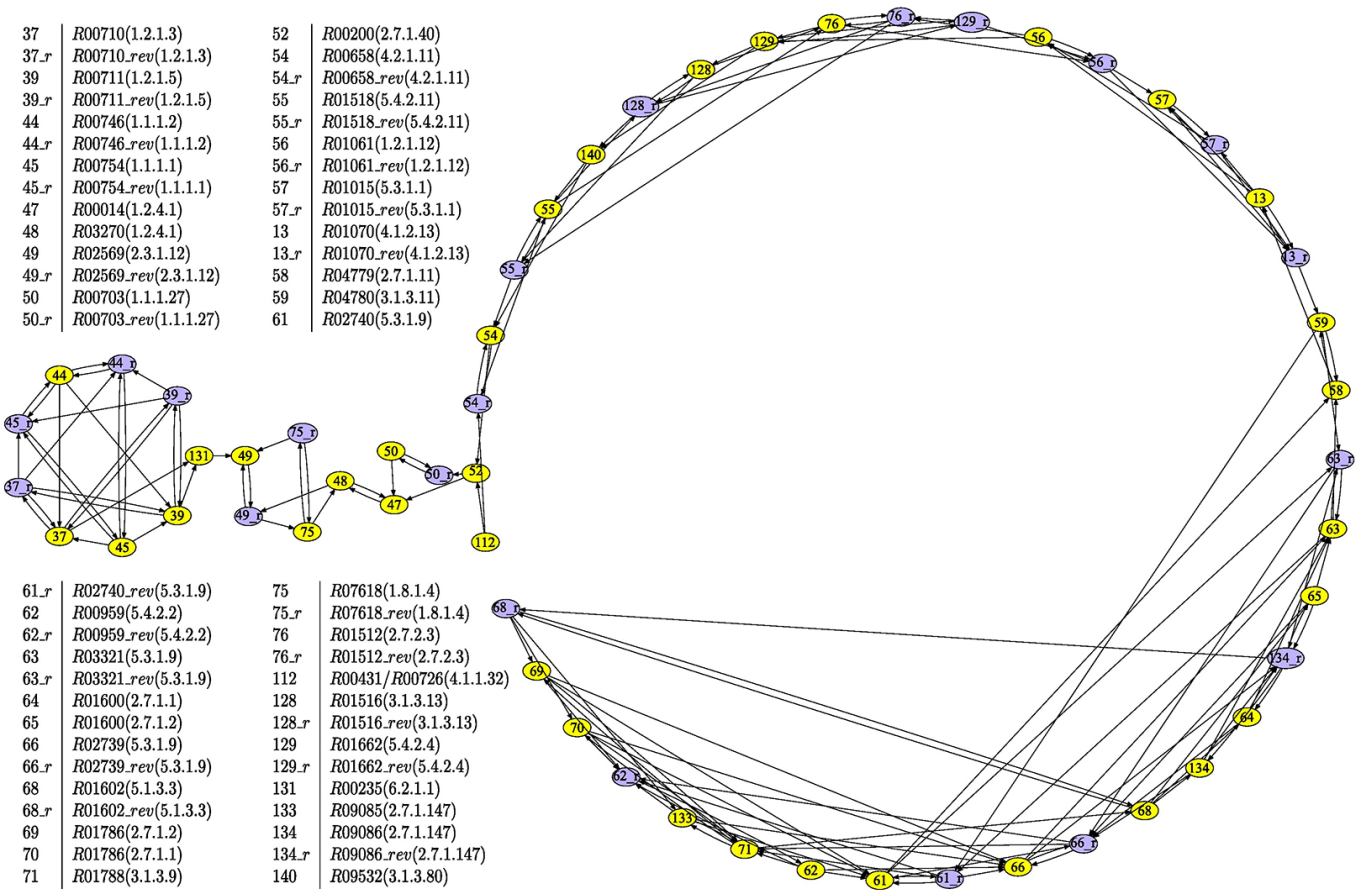
The reaction graph corresponding to the metabolic pathway of the glycolysis in *Homo sapiens*. It shows the reaction graph corresponding to the metabolic pathway depicted on the left side of Fig 4. The nodes in this graph are the reactions of the pathway, depicted in blue nodes are the reverse of a reversible reaction and in yellow the reaction itself.

**Fig 3:**
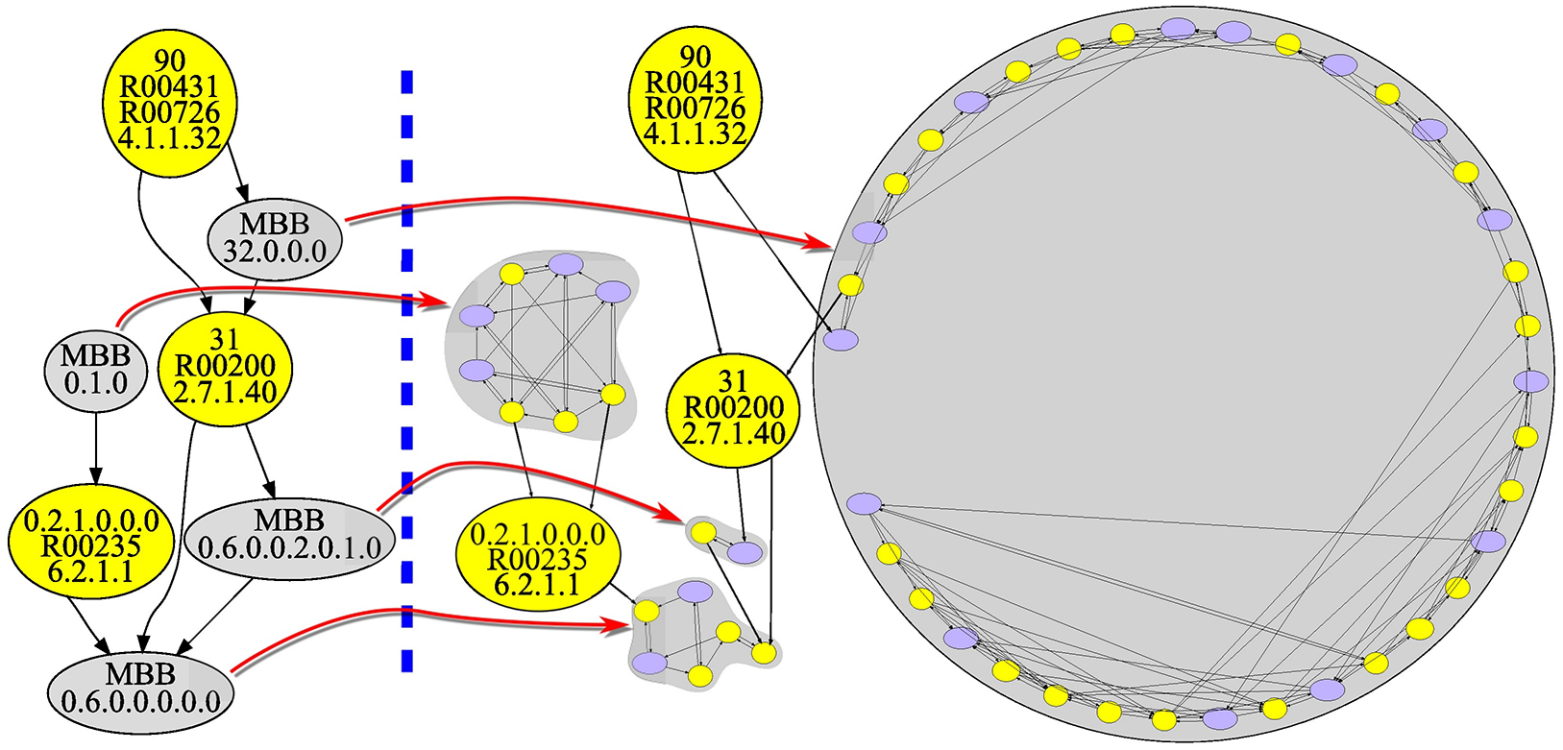
Relation between the reaction graph of the glycolysis in *Homo sapiens* and its corresponding m-DAG. It shows the relation between the reaction graph corresponding to the glycolysis pathway in *Homo sapiens* and its corresponding m-DAG. The m-DAG has seven nodes which are the corresponding MBBs in the reaction graph. Notice that three of them are yellow nodes, that is, a MBB with only one reaction, and four of them are grey nodes which are MBBs with more than one reaction. In Table 1 we list the reactions in every MBB.

Thus, in Fig 2 we show the reaction graph corresponding to the glycolysis pathway in *Homo sapiens* (http://www.genome.jp/kegg-bin/show_pathway?hsa00010) and in Fig 3 the relation between the reaction graph and its corresponding m-DAG.

In this case, the m-DAG that we obtain has seven MBBs, three of them with only one reaction and four of them with more than one reaction. The biggest one has 23 reactions two of them catalyzed by more than one enzyme, while the smallest one has only 2 reactions, *R*00703 and its reverse. As far as the arcs go, if we consider for instance the arc in the reaction graph from *R*00200 to *R*00703, then, there is also an arc in the m-DAG between the MBB 31 consisting only in *R*00200 and the MBB containing *R*00703, which is MBB 0.6.0.0.2.0.1.0. In Table 1, we show the collection of MBBs obtained from the reaction graph in Fig 2. As far as the essential reactions goes, a simple look at the m-DAG shows that there are two essential reactions, and among them, the essential reaction *R*00200 appears to be crucial to preserve the connectivity of the network, since it connects MBB 32.0.0.0 with MBB 0.6.0.0.0.0.0 which have 21 and 6 reactions respectively. Actually, the detailed study of the glycolysis pathway from the KEGG database that we carried on, reported in the next section, shows that the essential reaction *R*00200 is present in any organism performing the glycolysis pathway, which also entails that the enzyme catalyzing this reaction is a key enzyme.

**Table 1:** Collection of the MBBs in the reaction graph of the glycolysis in *Homo sapiens*.

Metabolic Building Blocks	Reactions
MBB 90	*R*00431/*R*00726
MBB 32.0.0.0	*R*00658^r^, *R*00959^r^, *R*01015^r^, *R*01061^r^, *R*01070^r^, *R*01512^r^, *R*01516^r^, *R*01518^r^, *R*01600^+^, *R*01602^r^, *R*01662^r^, *R*01786^+^, *R*01788, *R*02739^r^, *R*02740^r^, *R*03321^r^, *R*04779, *R*04780, *R*09085, *R*09086^r^, *R*09532
MBB 31	*R*00200
MBB 0.1.0	*R*00710^r^, *R*00711^r^, *R*00746^r^, *R*00754^r^
MBB 0.6.0.0.2.0.1.0	*R*00703^r^
MBB 0.2.1.0.0.0	*R*00235
MBB 0.6.0.0.0.0.0	*R*00014, *R*02569^r^, *R*03270, *R*07618^r^

Note: The ^r^ means that the corresponding reaction is reversible, and the ^+^ means that more than one enzyme catalyzes the reaction. Notice that in the reaction graph there is one different node for each enzyme catalyzing the reaction.

**Fig 4:**
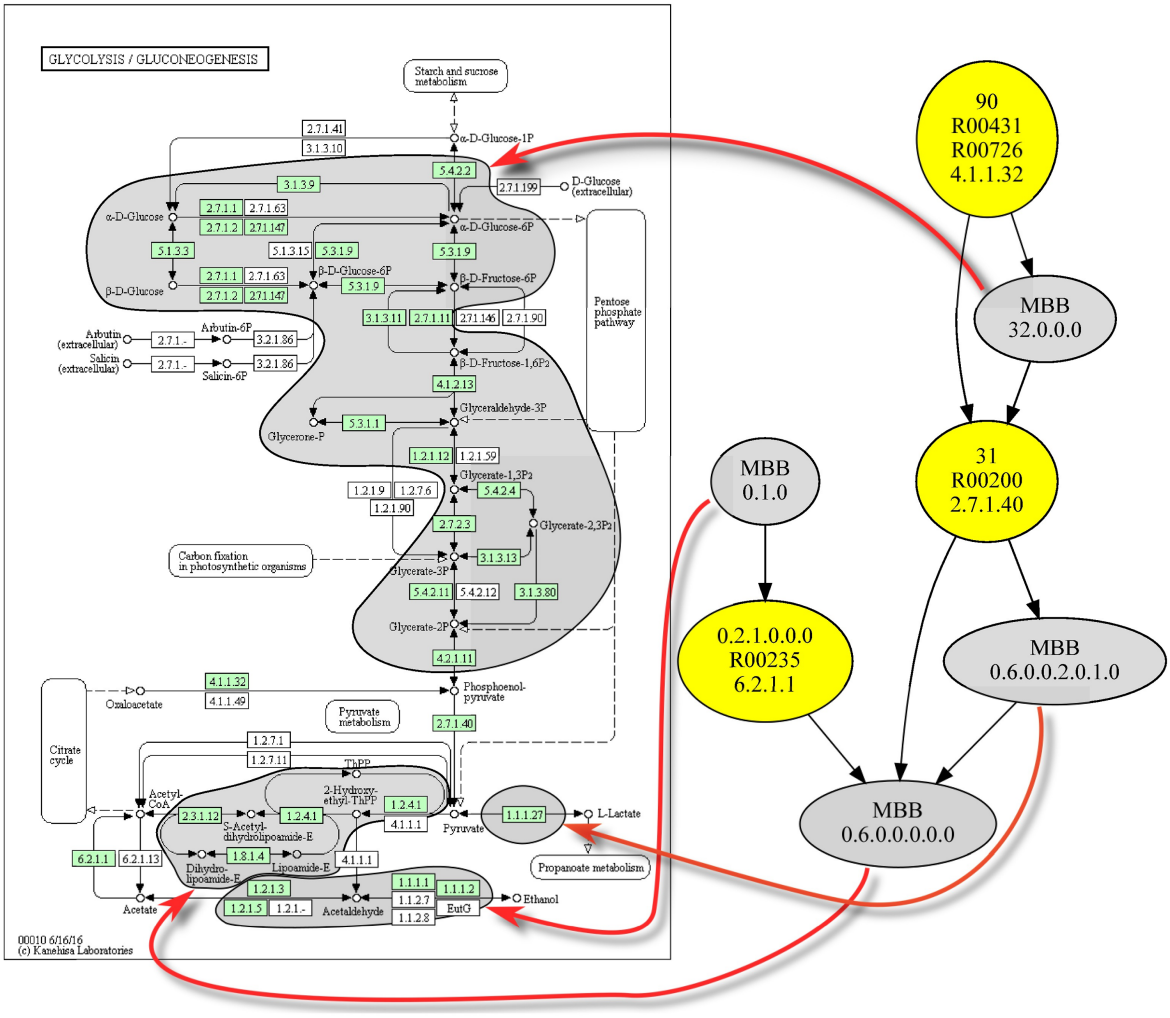
Relation between the *Homo sapiens* glycolysis pathway and its corresponding m-DAG. On the right is depicted the m-DAG corresponding to the *Homo sapiens* glycolysis pathway. For every MBB we show their corresponding reactions over the glycolysis pathway depicted on the left, as it is shown in the KEGG database.

Finally, since some readers are probably used to the glycolysis pathway description depicted in the KEGG database, we also show in Fig 4 the relation between the glycolysis pathway and the obtained m-DAG in *Homo sapiens*.

### Algorithm implementation

In order to implement the methodology proposed in this paper, we consider Tarjan’s strongly connected components algorithm [23], which is based on a depth first traversal of a directed graph. The idea is to visit all the nodes of the directed graph as follows: starting from any given one, the algorithm looks for nodes such that there exists an arc starting from the already discovered nodes and ending to an undiscovered one. The discovered nodes are stacked at the moment they are found, but they are popped out from the stack only when it is sure they belong to a new strongly connected component, that is, the whole strongly connected nodes are popped out at the same time.

Therefore, as the Tarjan’s algorithm is a variation of the depth first search algorithm, the overall complexity of this algorithm is *O*(|*R*| + |*E*|) where |*R*| is the number of nodes in the graph, in this case the number of reactions, and |*E*| is the number of arcs. The input in Tarjan’s algorithm is a directed graph, the reaction graph, while its output is a set of strongly connected components, the MBBs.

Once the MBBs have been found, the m-DAG is built by connecting each strongly connected component *MBB*_*i*_ with those other, *MBB*_*j*_, such that there exists at least one node in *MBB*_*i*_ with one or more arcs to nodes belonging to *MBB*_*j*_. The overall algorithm to do this process is shown in Algorithm 1.

**Algorithm 1**. **Algorithm to build the m-DAG from the Strongly Connected Components**.

**for all** *MBB*_*i*_ ∈ StronglyConnectedComponents **do**

 *neighbours*(*MBB*_*i*_) = ∅

 **for all** *n* ∈ *MBB*_*i*_ **do**

  **if** ∃ *m* ∈ *MBB*_*j*_|*n* → *m* ∧ *MBB*_*j*_ ≠ *MBB*_*i*_ **then**

   *neighbours*(*MBB*_*i*_) = *neighbours*(*MBB*_*i*_) ∪ {*MBB*_*j*_}

  **end if**

 **end for**

 **for all** *MBB*_*k*_ ∈ *neighbours*(*MBB*_*i*_) **do**

  add an arc between *MBB*_*i*_ and *MBB*_*k*_

 **end for**

**end for**

The result of this algorithm is a m-DAG whose nodes are the MBBs and whose arcs are their relations. In order to differentiate the MBBs, each one has been labelled with a number. This number corresponds with the one assigned by Tarjan’s algorithm. Thus, depending on the way the input graph is introduced to the algorithm, the numbers assigned may change.

Moreover, since many studies involves more than one species, in order to compare their m-DAGs, it is useful to unify their MBBs labels, because to compare two m-DAGs we have to compare their MBBs. If those MBBs have an identification describing their characteristics it is easy to differentiate them and also to see their similarities. In order to obtain those identifiers, a list of all different strongly connected components is built, each one has its list of reactions and also its unique identifier. Two MBBs are the same if their reaction lists are equal, and they are different if one of them contains some reaction the other doesn’t have. It is also possible that one reaction list is a subset of the other. Thus, the unique identifier of each MBB must reflect these three cases: if one MBB is a subset of another, its identifier has a prefix corresponding with the largest one, if two MBBs are different they have a completely different identifier, otherwise they have the same identifier.

To obtain this list of MBBs we start building the m-DAG of a *reference pathway* which is constructed, in the same way as in KEGG database, as the union of all reactions in every input reaction graph. The m-DAG obtained from the *reference pathway* is called a reference m-DAG. The set of reactions of every MBB appearing in a m-DAG of an organism is a subset of the set of reactions of a MBB from the reference m-DAG. The identifiers of the MBBs from the reference m-DAG will be the base of the identifiers of the MBBs of every m-DAG.

Prior to describe the algorithm we have devised to build the identifiers, we have to introduce some notation:

- react(*MBB*) represents the set of the reactions belonging to MBB.
- id(*MBB*) is the identifier of every *MBB*. Each MBB have an unique identifier in order to be able to differentiate it among the others.
- ref-DAG is the m-DAG obtained from the reference pathway. Since it is the most generic m-DAG, every reaction in an input reaction graph belongs to a MBB in the ref-DAG.
- subsets(*MBB*) represents the set of the *MBB*_*i*_, such that react(*MBB*_*i*_) ⊆ react(*MBB*).

Thus, the algorithm used to build the list of MBBs, *MBB-list*, is sown in Algorithm2.

**Algoritme 2**. **Algorithm used to build the list of MBBs**.

*MBB-list* ← ∅

**for all** *MBB*_*j*_ ∈ *m* − *DAG* **do**

 **if** ∃ *MBB*_*i*_ ∈ *MBB-list* | react(*MBB*_*j*_) = react(*MBB*_*i*_) **then**

  id(*MBB*_*j*_) ← id(*MBB*_*i*_)

 **else**

  **if** ∃ *MBB*_*i*_ ∈ ref-DAG | react(*MBB*_*i*_) = react(*MBB*_*j*_) **then**

   id(*MBB*_*j*_) ← next_identifier(subsets(*MBB*_*i*_))

   subsets(*MBB*_*i*_) ← subsets(*MBB*_*i*_) ∪ {*MBB*_*j*_}

  **else**

   **if** ∃ *MBB*_*i*_ ∈ ref-DAG | react(*MBB*_*j*_) ⊂ react(*MBB*_*i*_) **then**

    *MBB*_*s*_ → smallest *MBB* | *MBB*_*s*_ ∈ subsets (*MBB*_*i*_) ∧ *MBB*_*j*_ ⊂ *MBB*_*s*_

    subsets(*MBB*_*s*_) ← subsets(*MBB*_*s*_) ∪ {*MBB*_*j*_}

    id(*MBB*_*j*_) ← next_identifier(subsets(*MBB*_*s*_))

   **else**

    id(*MBB*_*j*_) ← new identifier

   **end if**

  **end if**

  *MBB-list* ← *MBB-list* ∪ {*MBB*_*j*_}

 **end if**

**end for**

Now, we proceed to relabel the nodes in the m-DAG for each species considered. For every m-DAG we compare its MBBs with those of the list. Let *MBB*_*i*_ be a MBB of the considered m-DAG. If there is a coincidence between *MBB*_*i*_ and a *MBB*_*j*_ in the list, we relabel *MBB*_*i*_ with the identifier of the *MBB*_*j*_. If there exist a *MBB*_*j*_ in the list such that react(*MBB*_*i*_) ⊂ react(*MBB*_*j*_) then id(*MBB*_*j*_) is a prefix of the identifier of *MBB*_*i*_. If there exists *MBB*_*j*_ in the list such that react(*MBB*_*j*_) ⊂ react(*MBB*_*i*_) then *MBB*_*j*_ is relabeled using id(*MBB*_*i*_) as a prefix and all the MBBs in subsets(*MBB*_*j*_) are relabeled accordingly. Otherwise *MBB*_*i*_ gets an new identifier not existing in the list.

**Fig 5:**
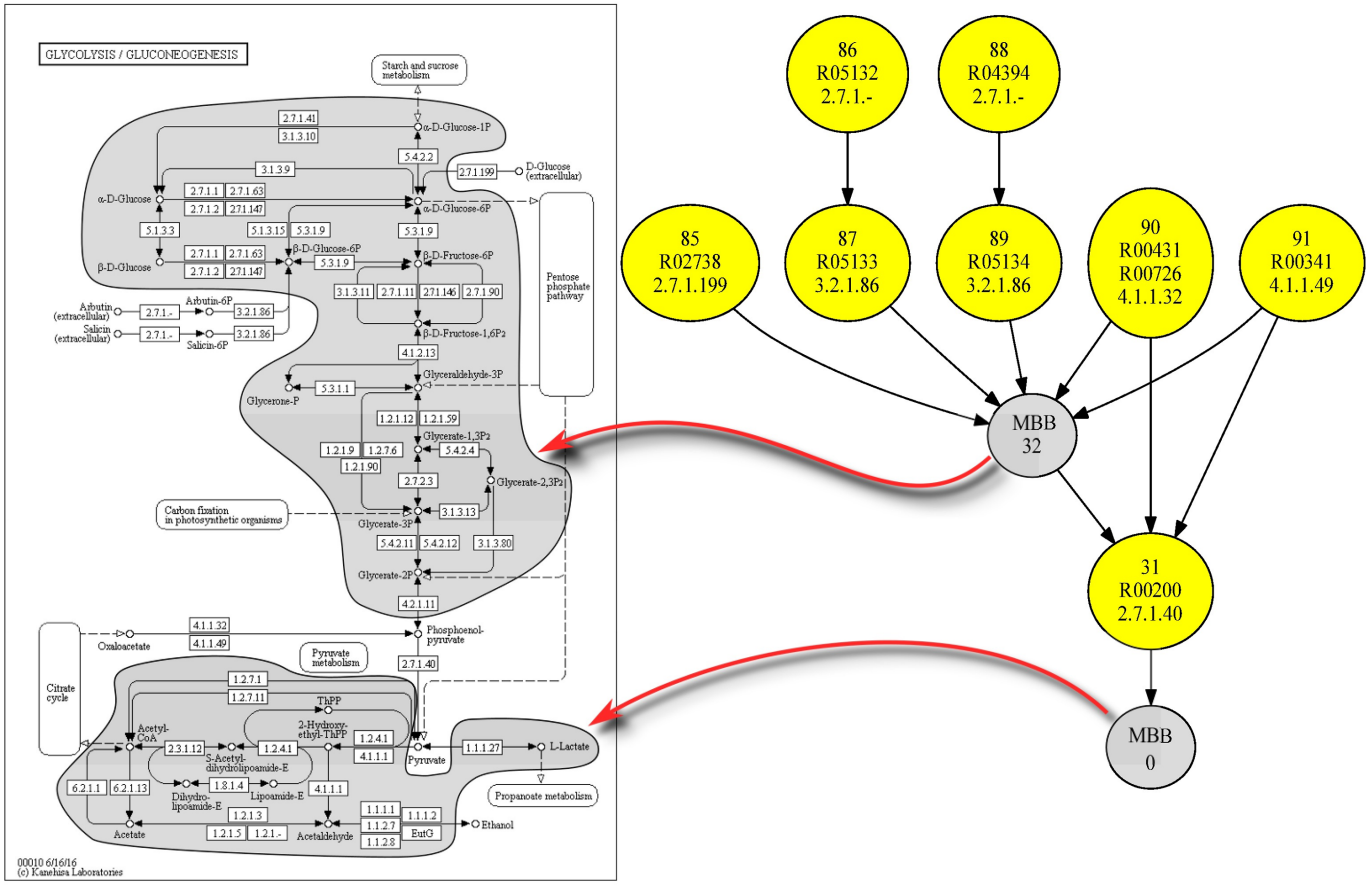
The glycolysis reference m-DAG. This figure shows the glycolysis reference m-DAG associated to the glycolysis reference pathway in the KEGG database. We again show the relation between the MBBs and their corresponding reactions over the reference glycolysis pathway depicted on the left.

In this way all the m-DAGs and also all the MBBs are comparable. For instance, as can be seen in Table 1, the MBBs have the identifiers: 90, 32.0.0.0, 31, 0.1.0, 0.6.0.0.2.0.1.0, 0.2.1.0.0.0 and 0.6.0.0.0.0.0. In Fig 5 there is a representation of the reference m-DAG corresponding to the glycolysis pathway. In this reference m-DAG there are also MBBs with the identifiers 90 and 31, which means that they are exactly the same MBBs that appear in the m-DAG of *Homo sapiens*. The MBB with identifier 32.0.0.0 has a prefix, 32, which indicates that the reactions of this MBB are all in the MBB with the identifier 32 in the reference m-DAG. The same occurs with the MBB with identifiers 0.1.0, 0.6.0.0.2.0.1.0, 0.2.1.0.0.0 and 0.6.0.0.0.0.0, whose reactions belong to MBB 0 in the reference m-DAG. Furthermore, with this MBB labeling, we can easily see that there is more similarity between MBB 0.6.0.0.2.0.1.0 and MBB 0.6.0.0.0.0.0 than for instance to MBB 0.1.0.

The presented algorithm is fast enough to be executed in a desktop or laptop computer. For example, in a 2.6 GHz Intel i7-6700HQ the average time needed to build a m-DAG for the purine metabolism is 0.63 seconds for each organism in the KEGG database. In the case of the glycolysis metabolism the average time needed to build a m-DAG is 0.03 seconds for each organism.

In http://bioinfo.uib.es/metabolomics/supplementary_material/ some supplementary material can be found as well as a web-based tool to give access to the researchers to our generated data.

## Results and discussion

In order to test the convenience of using m-DAGs to model and analyze metabolic networks, we considered the information available in the KEGG database corresponding to the glycolysis and the purine metabolic pathways. We decided to consider the glycolysis pathway since it is an ancestral set of chemical reactions present in almost every organism. On its turns, the purine pathway is also present in every organism and it is big enough to test the advantages of our methodology. Thus, with the information available on April 2016 in the KEGG database with 4158 different organisms, we ended up with 1653 different glycolysis pathways from 4140 organisms. These 1653 pathways were distributed in the six kingdoms defined in the KEGG database as suggested also by [24, 25]. The pathways’ distribution was: 33 from Animalia, 10 from Plantae, 27 from Fungi, 31 from Protista, 1420 from Bacteria and 132 from Archaea. Surprisingly, from 1653 different pathways, only 2 were mixed, the first one had 1 Plant and 13 Fungi, therefore we considered it in the Fungi distribution, and the second one had 1 Protists and 1 Bacterium, which we counted as both. We can not conclude if the mixture is due to a lack of information in the data or it is a biological metabolic variant. As far as the purine metabolism pathway goes, again with the information available on April 2016, we ended up with 2407 different pathways from 4158 organisms. In this case, the pathways distribution was: 76 from Animalia, 34 from Plantae, 81 from Fungi, 42 from Protista, 2019 from Bacteria and 145 from Archaea, so that every pathway was entirely classified in one kingdom.

### Metabolic networks analysis using m-DAGs

#### Glycolysis metabolism pathway test

We computed the 1653 m-DAGs from these 1653 different pathways and we analyzed them. From these 1653 m-DAGs, we obtained 299 MBBs. Tables in S1 and S5 Tables in the supporting information files show for every organism all the information of these MBBs. We also computed the m-DAG associated to the reaction graph of the reference pathway, reference m-DAG for short, which is shown in Fig 5. As we can observe there, the reference m-DAG has only 10 nodes, that is, 10 MBBs, three of them are essential reactions and only two of them are MBBs with more than one reaction. Notice that the number of nodes in the reference m-DAG with respect to the number of nodes in the reference reaction graph has been considerably reduced, which clarifies the metabolic network composition and allows us, not only to understand the entire network topology, but also to compare them easily. Table in S12 shows all the obtained information from the KEGG database for the glycolysis pathway.

**Fig 6:**
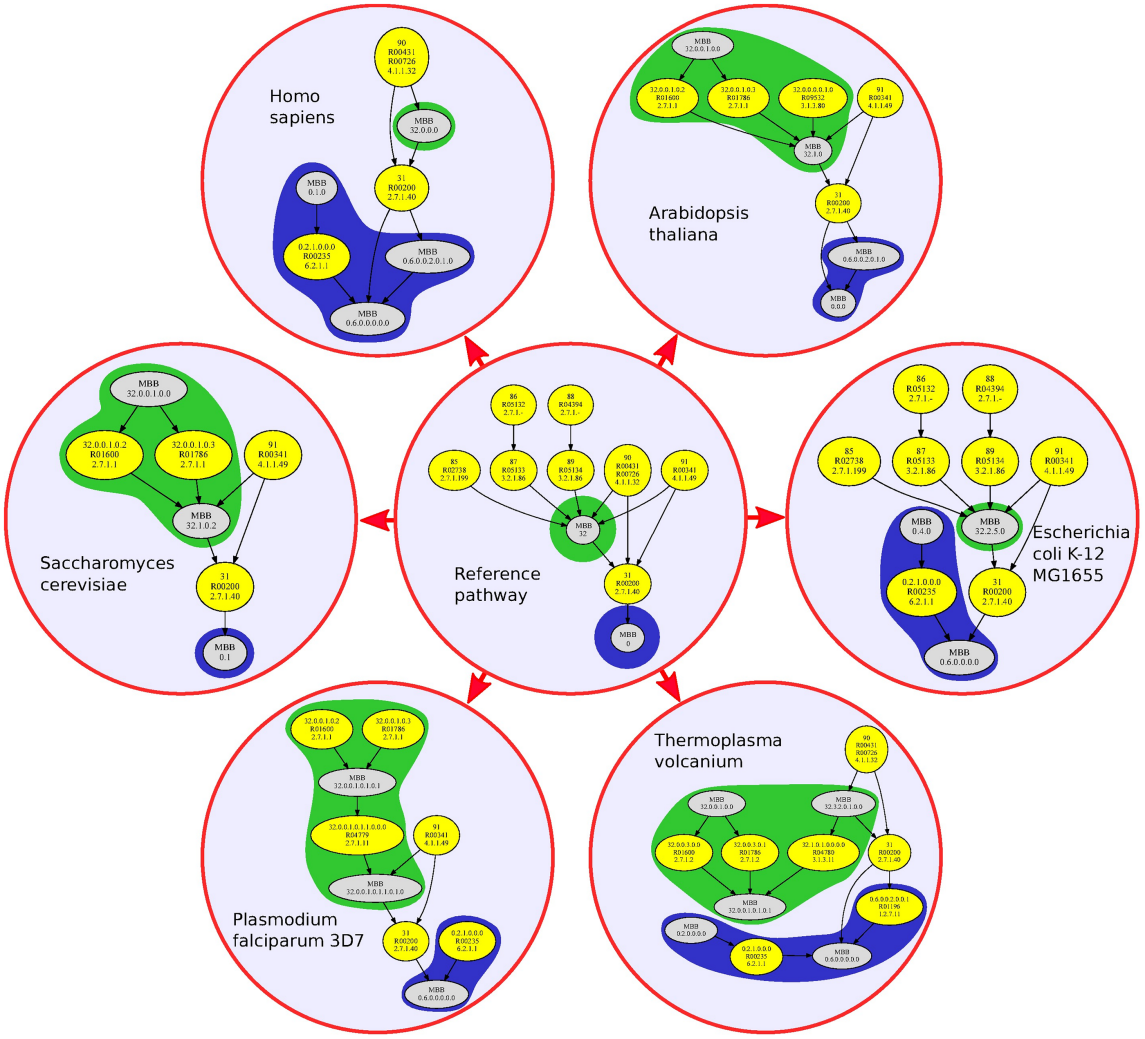
Relation between the reference m-DAG and the m-DAGs of six organisms from each kingdom. This figure shows the m-DAGs of six organisms, one in each kingdom and their relation with the reference m-DAG (in the center). The two MBBs in the reference m-DAG with more than one reaction are depicted in green and blue. With the corresponding color (green or blue) in the background, we show the inclusion of the MBBs of every organism into the MBBs in the reference m-DAG.

To study the biological information captured by the MBBs defined in this paper, we took advantage of the fact that the glycolysis pathway of any organism in the KEGG database is included in the glycolysis reference pathway. Thus, the corresponding reaction graphs also preserve this inclusion and, for every organism, its MBBs are also included in one MBB from the reference m-DAG (see Fig 6). Therefore, the information provided by the MBBs in the reference m-DAG (in the center of Fig 6) is very valuable. In fact, every essential reaction in the reference pathway is also an essential reaction in the glycolysis pathway of any organism, provided that the reaction is present in that organism. This fact renders the essential reactions in the reference m-DAG as *key* MBBs for the glycolysis pathway of any organism, because they fix the connectivity in every glycolysis pathway.

In the present study we have found two essential processes: *(i)* the MBB with a direct arc to reaction *R*00200, and, *(ii)* the MBB with a direct arc from reaction *R*00200. Hence, *R*00200 appears to be a crucial essential reaction that has been conserved among all organisms with glycolysis and has been preserved in every evolution step, since there is only one way to produce pyruvate from phosphoenol-pyruvate, and it is through reaction *R*00200 catalyzed by the enzyme 2.7.1.14 corresponding to a *pyruvate kinase*. As expected, few exceptions have been found, and only 18 organisms out of 4158 do not follow this rule. These 18 organisms are Nanoarchaeota, Alphaproteobacteria, Betaproteobacteria and Gammaproteobacteria (See Table 2). For instance, as we can found in the Bergey’s manual [26], Betaproteobacteria are unable to assimilate any of the carbohydrates or polyols and can grow only on amino acids and organic acids. This indicates that one or more steps of glycolysis are absent in both organisms. Therefore, we claim that we found out that these three MBBs (the MBB with a direct arc to reaction *R*00200, the MBB consisting on reaction *R*00200 and the MBB with a direct arc from reaction *R*00200) can be considered as the *core* of the glycolysis pathway.

**Table 2:** Classification of the eighteen species without reaction *R*00200.

Category	# species
Unclassified Archaea	1
Nanoarchaeota	1
Alphaproteobacteria	4
Betaproteobacteria	5
Gammaproteobacteria—Others	7

In order to reinforce the concept up to now investigated, in Fig 6 we can observe easily the network and biological information captured by the MBBs. We considered the m-DAGs corresponding to the glycolysis of the following organisms, *Homo sapiens*, *Arabidopsis thaliana*, *Saccharomyces cerevisiae*, *Plasmodium falciparum*, *Escherichia coli* and *Thermoplasma volcanium* belonging to the kingdoms, Animalia, Plantae, Fungi, Protista, Bacteria/Eubacteria, and Archaea/Archaeabacteria, respectively. We observe there that all the organisms has the essential reaction *R*00200, which appears to be crucial in the glycolysis pathway. We can also see how the MBBs are distributed in the two processes mentioned above. In green we show the MBBs with a direct arc to reaction *R*00200 and in blue the MBBs with a direct arc from reaction *R*00200.

In the same way, we also developed a similar analysis considering again the six kingdoms. In this case, the purpose was to find out, with the information provided by the m-DAGs, what we called the kingdom reference m-DAG, which is the reference m-DAG for each kingdom. (See S3, S4, S5, S6, S7 and S8 Figs in the supporting information files.) To obtain the kingdom reference m-DAG, we first considered all the MBBs from every m-DAG belonging to the same kingdom. We decided to remove the glycolysis pathway of *Pyrodictium delaneyi*, which has a pathway alternative from glycolysis due to the fact that this organism habits in deep-sea hydrothermal vent sulfide chimneys [27].

**Fig 7:**
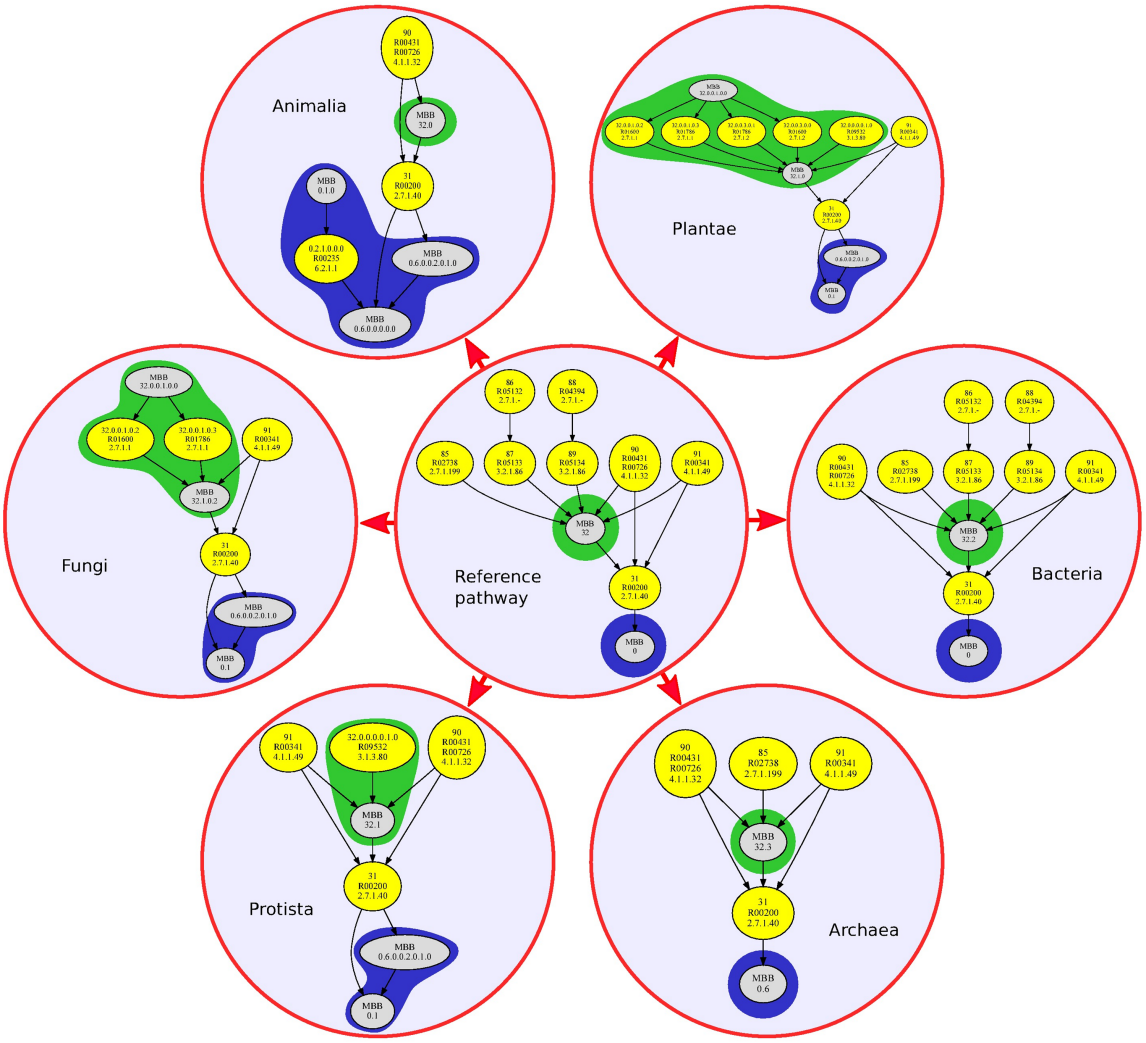
The glycolysis kingdom reference m-DAGs. This figure shows the kingdom reference m-DAGs obtained for the six kingdoms as well as their relation with the reference m-DAG in the center of the figure.

Recall that, every MBB belongs to one of the following classes: *(i)* the MBB with a direct arc to reaction *R*00200, *(ii)* the MBB with a direct arc from reaction *R*00200 and *(iii)* the MBB that have only one reaction in the reference m-DAG. So, we considered the maximal MBBs in each class and thus we obtained the six kingdom reference m-DAGs shown in Fig 7. As we can see there, the kingdom reference m-DAGs are connected DAGs and they preserve the reference m-DAG’s topological structure. The MBB in the first class in the reference m-DAG (pointed out in green) is divided in seven MBBs in Plantae, in two MBBs in Protista and in four MBBs in Fungi. The MBB in the second class in the reference m-DAG (pointed out in blue) is divided in four MBBs in Animalia, in two MBBs in Plantae, in two MBBs in Protista and in two MBBs in Fungi. We can also observe that all the kingdom reference m-DAGs have the MBB consisting only in reaction *R*00200. As far as the other MBBs in the third class goes, the Animalia have only two MBBs in the third class, as well as the Plantae and the Fungi. The Bacteria have the same MBBs in the third class as in the reference m-DAG and the Protista have three MBBs in the third class. Notice that from the results obtained in this test, we can discriminate whether a specific reaction in the glycolysis pathway belongs to a kingdom. For instance, if enzyme 3.2.1.86 is present, we can guarantee that it belongs to an organism in the Bacteria kingdom. While instead, if enzyme 4.1.1.49 is present, we can guarantee that it does not belong to Animalia nor Archaea. In Table 3 we provide a list of the enzymes and the reactions they catalyze that discriminate the kingdoms. As can be seen, the same enzyme catalyzes two reactions that belong only to Animalia; another one catalyzes two reactions belonging only to Archaea. Six enzymes catalyzes eight reactions belonging only to Bacteria. Interestingly, no specific reactions have been found to belong only to Fungi, Plantae or Protista.

**Table 3:** Enzymes and reactions that discriminate the kingdoms in the glycolysis pathway.

Enzyme code	Enzyme name	Reactions	Kingdom
3.1.3.13	bisphosphoglycerate mutase	R01516	Animalia
5.4.2.4	bisphosphoglycerate mutase	R01662	Animalia
1.2.1.90	glyceraldehyde-3-phosphate dehydrogenase [NAD(P)+]	R01058/R10860	Archaea
1.2.1.-	aldehyde dehydrogenase	R00711^r^	Bacteria
1.1.2.7	methanol dehydrogenase (cytochrome c) subunit 1	R09127^r^	Bacteria
2.7.1.41	glucose-1-phosphate phosphodismutase	R00960	Bacteria
2.7.1.63	polyphosphate glucokinase	R20187, R20189	Bacteria
3.1.3.10	glucose-1-phosphatase	R00947	Bacteria
3.2.1.86	6-phospho-beta-glucosidase	R05133, R05134	Bacteria
	*No reaction appears only in this kingdom*.		Fungi
	*No reaction appears only in this kingdom*.		Plantae
	*No reaction appears only in this kingdom*.		Protista

Note: The ^r^ means that the corresponding reaction is reversible.

#### Purine metabolism pathway test

**Fig 8:**
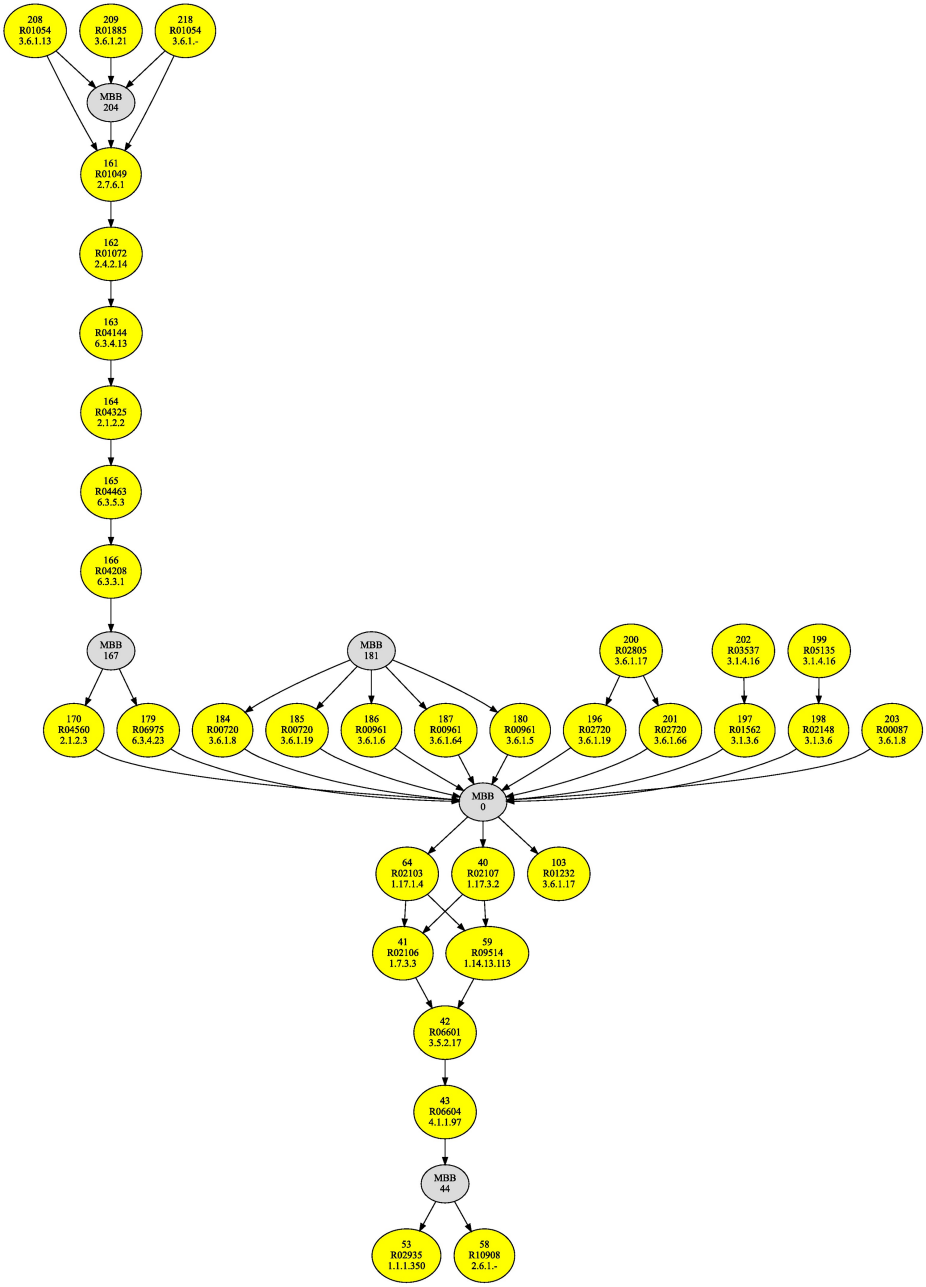
The purine metabolism reference m-DAG. This figure shows the purine metabolism reference m-DAG associated to the purine metabolism reference pathway in the KEGG database.

We computed the m-DAG from each different pathway in the KEGG database and we analyzed them. Recall that with the information available on April 2016, we ended up with 2407 different pathways from 4158 organisms. From these 2407 m-DAGs we obtained 1386 MBBs. Again, to study the biological information captured by the MBBs defined in this paper, we took advantage of the fact that the purine metabolism pathway of any organism in the KEGG database is included in the purine metabolism reference pathway. Thus, the corresponding reaction graphs also preserve this inclusion and, for every organism, its MBBs are also included in one MBB from the reference m-DAG. Therefore, the information provided by the MBBs in the reference m-DAG is very valuable. Indeed, we computed the reference m-DAG, which is shown in Fig 8, and we obtain 38 nodes, that is, 38 MBBs in the purine metabolism reference pathway. Among them, 5 MBBs have more than one reaction and 33 have only one reaction, of which only eight are essential reactions. If we try to compare now the m-DAG obtained in *Homo sapiens* and the reference m-DAG, we can easily see that there is a sequence of six essential reactions present in both DAGs. This sequence of essential reactions produces the 5-Aminoimidazole-4-carboxamide ribonucleotide (AICAR) from the Ribose 5-phosphate. The fact that this sequence is present in the reference m-DAG entails that every organism with the purine metabolism pathway has exactly the same sequence. As we can also observe in the reference m-DAG, there are two MBBs with only one reaction, MBB 170 and MBB 179 connecting the sequence of essential reactions with MBB 0, a MBB with 139 reactions conducting to the Purines.

Therefore, in this study we have found the following essential processes: *(i)* the sequence of essential MBBs that produces the (AICAR) from the Ribose 5-phosphate and, *(ii)* MBB 0 with all reactions conducting to the Purines, that is, either Adenine or Guanine. These two processes could be considered the *core* of the purine metabolism pathway for all the organisms in KEGG.

Analogous to the glycolysis pathway, we also developed a similar analysis considering again the six kingdoms. We computed the reference m-DAG for each one called again the kingdom reference m-DAG (shown in S9, S10, S11, S12, S13 and S14 Figs) and we looked for those MBBs that discriminate among them. As expected, we obtain that every kingdom contains the two essential processes defined as the core of the purine metabolism pathway, and also, each kingdom has a set of different MBBs connected to these processes. In Table 4 we show the MBBs that discriminate the kingdoms; that is, the MBBs that are not present in all kingdoms. In Table 5 we show the enzymes and reactions corresponding to the MBBs in Table 4.

**Table 4:** MBBs that discriminate the kingdoms in the purine metabolism pathway.

	Animalia	Plantae	Protista	Fungi	Bacteriae	Archaea
*MBB 179*					✓	✓
*MBB 184*					✓	
*MBB 185*	✓	✓	✓	✓	✓	
*MBB 186*	✓	✓	✓		✓	
*MBB 187*	✓				✓	
*MBB 180*	✓	✓	✓	✓	✓	
*MBB 196*	✓	✓	✓	✓	✓	
*MBB 201*					✓	✓
*MBB 197*					✓	✓
*MBB 198*					✓	✓
*MBB 203*					✓	

**Table 5:** Enzymes and reactions that discriminate the kingdoms in the purine metabolism pathway.

	Reaction	Enzyme	Enzyme
MBB 179	*R*06975	6.3.4.23	formate—phosphoribosylaminoimidazolecarboxamide ligase
MBB 184	*R*00720	3.6.1.8	ATP diphosphatase
MBB 185	*R*00720	3.6.1.19	nucleoside-triphosphate diphosphatase
MBB 186	*R*00961	3.6.1.6	nucleoside diphosphate phosphatase
MBB 187	*R*00961	3.6.1.64	inosine diphosphate phosphatase
MBB 180	*R*00961	3.6.1.5	apyrase
MBB 196	*R*02720	3.6.1.19	nucleoside-triphosphate diphosphatase
MBB 201	*R*02720	3.6.1.66	XTP/dITP diphosphatase
MBB 197	*R*01562	3.1.3.6	nucleoside diphosphate phosphatase
MBB 198	*R*02148	3.1.3.6	nucleoside diphosphate phosphatase
MBB 203	*R*00087	3.6.1.8	ATP diphosphatase

Notice the importance of MBB 170, corresponding to *R*04560. It is a crucial essential reaction in Animalia, Plantae, Protista and Fungi that connects the two processes described as the core of the purine metabolism pathway. On the other hand, MBB 179, corresponding to *R*06975 is only present in one member of the Bacteriae (genus Peribacter) and in more than hundred members of the Archaea. Thus, we can conclude that in Procaryotes two different ways to connect the two essential processes have been found, in contrast to the only one way found in Eukaryotes. Interestingly, MBB 201, MBB 197, MBB 198, and MBB 203 are only present in Procaryotes.

### Glycolysis evolution

The second test performed to study the convenience of using m-DAGs to model metabolic networks, was to extend the MBBs information to obtain an overall clustering of all organisms with glycolysis and provide some contribution to answer the question, *does metabolomics meet genomics?*

To obtain a clustering of all organisms with glycolysis, we first defined a similarity measure between two m-DAGs based on the similarity of their MBBs. To define a similarity score of two MBBs, we first defined a reactions similarity score as it was done in [12]. Let *R*_*i*_ = (*I*_*i*_, *E*_*i*_, *O*_*i*_) and *R*_*j*_ = (*I*_*j*_, *E*_*j*_, *O*_*j*_) be two reactions, their similarity score *SimReact*(*R*_*i*_, *R*_*j*_) is given by the following formula [13]:

$$\begin{array}{c} SimReact\left(R_{i},R_{j}\right)=SimEnz\left(E_{i},E_{j}\right)\cdot w_{e}+SimComp\left(I_{i},I_{j}\right)\cdot w_{i}+SimComp\left(O_{i},O_{j}\right)\cdot w_{o} \end{array}$$ (1)

where *SimEnz*(*E*_*i*_, *E*_*j*_) is the enzyme similarity between *E*_*i*_ and *E*_*j*_, *SimComp*(*I*_*i*_, *I*_*j*_) is the compound similarity between the substrates *I*_*i*_, *I*_*j*_ and *SimComp*(*O*_*i*_, *O*_*j*_) is the compound similarity between the products *O*_*i*_, *O*_*j*_. The parameters *w*_*e*_, *w*_*i*_ and *w*_*o*_ are fixed to *w*_*e*_ = 0.4 and *w*_*i*_ = *w*_*o*_ = 0.3 since, as stated in [13], they provide a good balance between enzymes and compounds (chemical substances).

For the enzyme and compound similarities in Eq (1) we made the following choices.

- For enzymes, we used the EC hierarchical similarity measure that is based on the comparison of the unique *EC number* (Enzyme Commission number) associated to each enzyme, which represents its catalytic activity. The EC number is a 4-level hierarchical scheme, *d*_1_.*d*_2_.*d*_3_.*d*_4_, developed by the International Union of Biochemistry and Molecular Biology (IUBMB) [28]. Enzymes with similar EC classifications are functional homologues but do not necessarily have similar amino acid sequences.
  Given two enzymes *e* = *d*_1_.*d*_2_.*d*_3_.*d*_4_ and $e^{\prime }=d_{1}^{\prime }.d_{2}^{\prime }.d_{3}^{\prime }.d_{4}^{\prime }$, their similarity *S*(*e*, *e*′) depends on the length of the common prefix of their EC numbers:
  $$S\left(e,e\prime \right)=\text{max}\left\{i=1,2,3,4:d_{j}=d_{j^{\prime }},j=1,\ldots ,i\right\}/4$$
  For instance, the similarity between *arginase* (*e* = 3.5.3.1) and *creatinase* (*e*′ = 3.5.3.3) is 0.75.
- For compounds, we used a similarity based on the similarity measure computed by the SIMCOMP (SIMilar COMPound) [29] tool. Given two compounds, the tool represents their chemical structure as graphs and outputs a measure of their maximal common substructure.
  Since a reaction may have more than one input (output) compound, we needed a way to combine the similarity between pairs of compounds computed by SIMCOMP. Given two sets *X* and *Y* of compounds, the score *SimComp*(*X*, *Y*) was computed by:
  – defining a complete bipartite graph in which the compounds in *X* and *Y* are nodes and the weight of each arc (*x*, *y*) ∈ *X* × *Y* is the similarity value of *x* and *y* computed by SIMCOMP;
  – applying the maximum weighted bipartite matching algorithm to the resulting graph to obtain the best match between *X* and *Y*;
  – summing the scores of the best match and dividing it by max{|*X*|, |*Y*|}.

Next, given two MBBs, *MBB*_1_ and *MBB*_2_ its similarity score, *S*_*mbb*_(*MBB*_1_, *MBB*_2_), was computed by:

- defining a complete bipartite graph in which the reactions in *MBB*_1_ and *MBB*_2_ are nodes and the weight of each arc (*R*_*i*_, *R*_*j*_)∈*MBB*_1_ × *MBB*_2_ is *SimReact*(*R*_*i*_, *R*_*j*_);
- applying the maximum weighted bipartite matching algorithm to the resulting graph to obtain the best match between *MBB*_1_ and *MBB*_2_;
- summing the scores of the best match and dividing it by max{|*MBB*_1_|, |*MBB*_2_|}.

Finally, the similarity measure between two m-DAG, *Sim*(*mD*_1_, *mD*_2_) was computed by:

- defining a complete bipartite graph in which the MBBs in *mD*_1_ and *mD*_2_ are nodes and the weight of each arc (*MBB*_*i*_, *MBB*_*j*_) ∈ *mD*_1_ × *mD*_2_ is *S*_*mbb*_(*MBB*_1_, *MBB*_2_);
- applying the maximum weighted bipartite matching algorithm to the resulting graph to obtain the best match between *mD*_1_ and *mD*_2_;
- summing the scores of the best match and dividing it by max{|*mD*_1_|, |*mD*_2_|}.

In this test, since we had already analyzed the glycolysis pathways from the KEGG database, we considered the information captured by the reference m-DAG and we again split the MBBs in three classes: *(i)* the MBB with a direct arc to reaction *R*00200, *(ii)* the MBB with a direct arc from reaction *R*00200 and *(iii)* the MBB with only one reaction in the reference m-DAG. Then, to obtain the complete bipartite graph in the previous computation, we only considered the similarity measure of two MBBs in the same class and the weight of an arc between two MBBs in different classes was set to zero.

Once the similarity measure between every pair of glycolysis m-DAG was computed, we converted the similarity score into the following distance measure:

$$\begin{array}{c} d\left(H_{1},H_{2}\right)=\sqrt{1-{\left(Score\left(H_{1},H_{2}\right)\right)}^{2}} \end{array}$$ (2)

**Fig 9:**
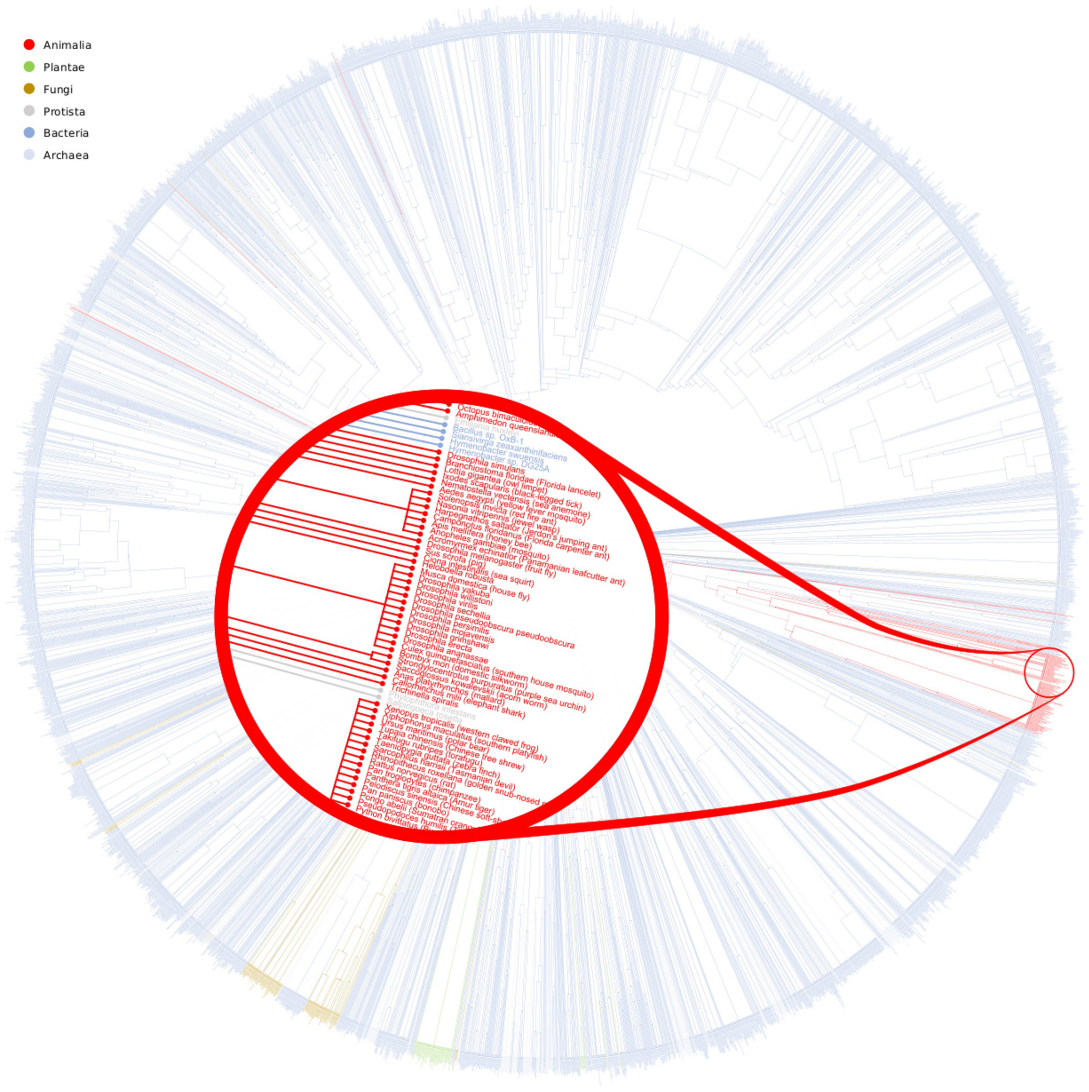
Glycolysis evolution. This figure shows the dendrogram obtained with the hierarchical clustering of the glycolysis pathway. Red, green, brown, grey, dark blue and light blue labels are m-DAGs in Animalia, Plantae, Fungi, Protista, Bacteria and Archaea, respectively. In S15 Fig we provide the vectorial format of this image for a better visualization.

Then, we performed the hierarchical clustering based on the distance measure above. To obtain the hierarchical clustering of the 1653 different m-DAGs we used the UPGMA method. In Fig 9 we show the results of this hierarchical clustering. Notice that there are 4140 different organisms represented in this dendrogram so the details are very small. However, we provide the vectorial format of this image in S15 Fig in the supporting information files for a better visualization, in such a way that the reader can magnify an area of the image to see the smallest details. If we try to understand those results as a glycolysis tree evolution, we can observe that, despite Bacteria, the other kingdoms are clustered together. Notice that, the amount of bacteria is considerably bigger than other kingdoms. Therefore, it is not surprising that they appear everywhere in the dendrogram. The differences detected inside a kingdom are a consequence of the adaptation of the organisms to the different habitats or to a divergence in the evolutionary processes.

### Purine metabolism evolution

**Fig 10:**
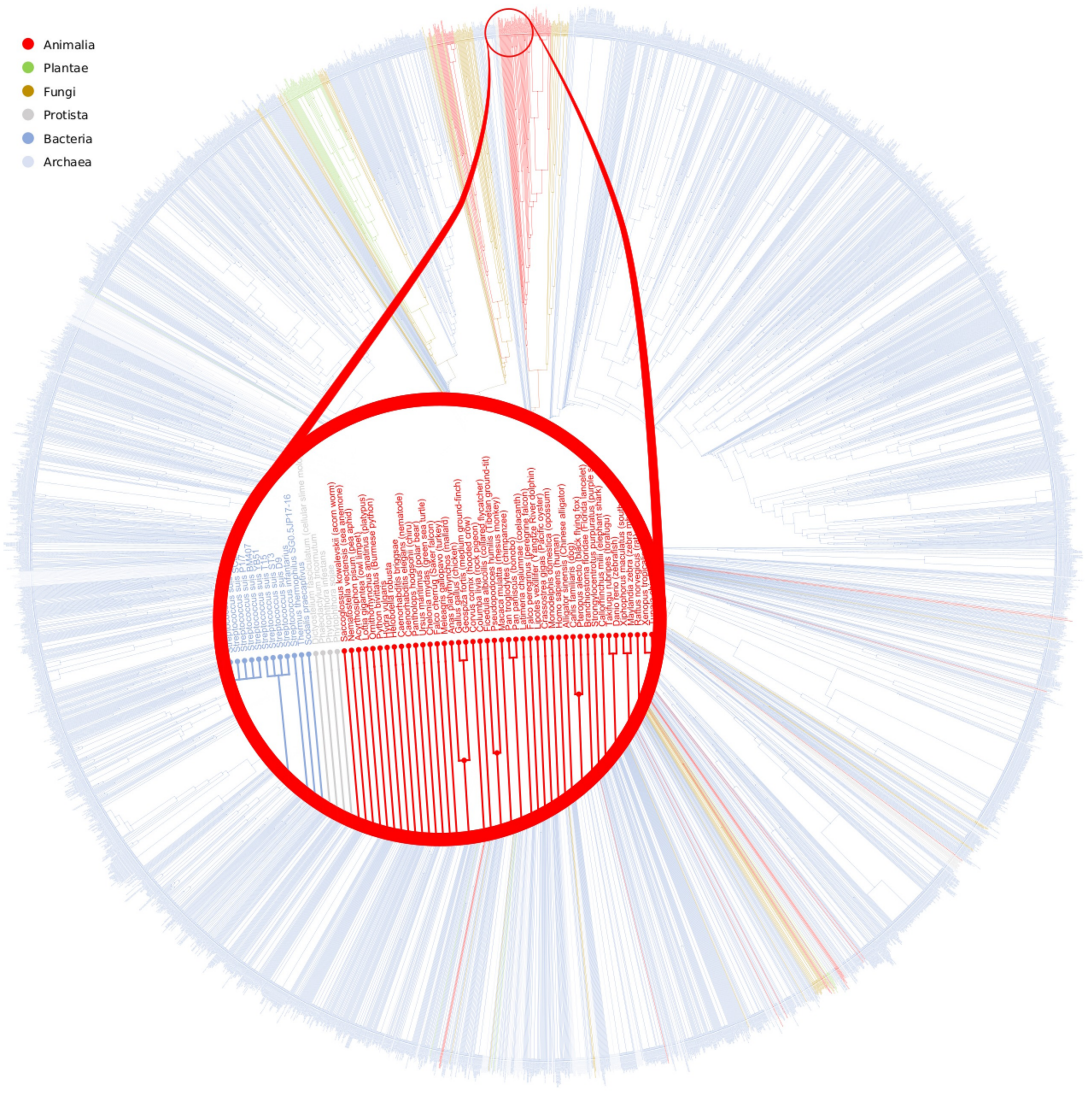
Purine metabolism evolution. This figure shows the dendrogram obtained with the hierarchical clustering of the purine metabolism pathway. Red, green, brown, grey, dark blue and light blue labels are m-DAG in Animalia, Plantae, Fungi, Protista, Bacteria and Archaea, respectively. In S16 Fig we provide the vectorial format of this image for a better visualization.

Again, the second test applied to the purine metabolism pathway to study the convenience of using metabolic DAGs to model metabolic networks, was to extend the MBBs information to obtain an overall clustering of all organisms, as it was done in the glycolysis test (See Fig 10). In this test, we obtain a similar results, that is, despite Bacteria, the other kingdoms are clustered together. And again, the differences detected inside a kingdom are a consequence of the adaptation of the organisms to the different habitats or to a divergence in the evolutionary processes.

## Conclusion

In this paper we introduce a new approach of metabolic networks modeling based on classical notions of graph theory, which applied to metabolic networks has been successful. Namely, we recover the notion of strongly connected components, that we call MBBs, to obtain a modularization of the network, called a m-DAG. One of the advantages of our methodology is to be general enough to perform for any database, although we run our tests using the data information in the KEGG database. We applied this methodology to two metabolic pathways to reinforce its utility. We considered the glycolysis (an energetic pathway) and the purine metabolism (essential for DNA) present in the living beings. The glycolysis is a simple pathway which allows us to visualize the relation between the pathway and the m-DAGs, while the purine metabolism is big enough to test the convenience of the reduction defined in this work. Using the m-DAG formalism, not only a considerable reduction on the size of the metabolic network has been obtained, which facilitates the network comprehension, but also a new concept, the MBBs has been introduced. From the information captured by the m-DAG and its corresponding MBBs, we find the *core* of the glycolysis pathway and detect some essential MBBs that reveal the *key* reactions in the pathway as well as the *core* of the purine metabolism pathway. Finally, the application of this methodology to both pathways reproduces the tree of life, supporting the utility of this research.

## Supporting information

S1 Table MBBs for the glycolysis pathway. A file containing the reactions and the amount of species in each kingdom that each MBB has in the glycolysis pathway.
(CSV)

S2 Table MBBs for the purine metabolism pathway. Same data as in the S1 Table but for the purine metabolism.
(CSV)

S3 TableMBB distances for the glycolysis.A file with the distances between every pair of MBBs in the glycolysis pathway based on the following distance measure: $d(MBB_{1},MBB_{2})=\sqrt{1-{\left(S_{mbb}(MBB_{1},MBB_{2})\right)}^{2}}$.(CSV)

S4 TableMBB distances for the purine.A file with the distances between every pair of MBBs in the purine metabolism pathway based on the following distance measure: $d(MBB_{1},MBB_{2})=\sqrt{1-{\left(S_{mbb}(MBB_{1},MBB_{2})\right)}^{2}}$.(ZIP)

S5 Table Glycolysis m-DAG summary. A file with the following information: for every glycolysis m-DAG, the reactions it has and its MBBs.
(CSV)

S6 Table Purine m-DAG summary. A file with the following information: for every purine metabolism m-DAG, the reactions it has and its MBBs.
(ZIP)

S7 Table m-DAG distances for the glycolysis. A file with the distances between every pair of m-DAGs, based on the similarity measure described in this paper.
(ZIP)

S8 Table m-DAG distances for the purine. A file with the distances between every pair of m-DAGs, based on the similarity measure described in this paper.
(ZIP)

S9 Table Glycolysis species per m-DAG. A file with the following information: for every m-DAG its amount of species in each kingdom.
(CSV)

S10 Table Purines species per m-DAG. A file with the following information: for every m-DAG its amount of species in each kingdom.
(CSV)

S11 Table m-DAG worksheet for glycolysis. A worksheet combining the m-DAG related files for an easy and human readable access.
(ZIP)

S12 Table MBB worksheet for glycolysis. A worksheet combining the MBB related files for an easy and human readable access.
(ZIP)

S13 Table m-DAG worksheet for purine metabolism. A worksheet combining the m-DAG related files for an easy and human readable access.
(XLSX)

S14 Table MBB worksheet for purine metabolism. A worksheet combining the MBB related files for an easy and human readable access.
(XLSX)

S1 Fig Glycolysis MBB dendrogram. A dendrogram obtained with the hierarchical clustering of the MBBs using the distances in S3 Table.
(PDF)

S2 Fig Purine MBB dendrogram. A dendrogram obtained with the hierarchical clustering of the MBBs using the distances in S4 Table.
(PDF)

S3 Fig Animalia reference m-DAG for glycolysis. A file with the Animalia kingdom reference m-DAG for the glycolysis pathway.
(PDF)

S4 Fig Plantae reference m-DAG for glycolysis. A file with the Plantae kingdom reference m-DAG for the glycolysis pathway.
(PDF)

S5 Fig Fungi reference m-DAG for glycolysis. A file with the Fungi kingdom reference m-DAG for the glycolysis pathway.
(PDF)

S6 Fig Protista reference m-DAG for glycolysis. A file with the Protista kingdom reference m-DAG for the glycolysis pathway.
(PDF)

S7 Fig Bacteria reference m-DAG for glycolysis. A file with the Bacteria kingdom reference m-DAG for the glycolysis pathway.
(PDF)

S8 Fig Archaea reference m-DAG for glycolysis. A file with the Archaea kingdom reference m-DAG for the glycolysis pathway.
(PDF)

S9 Fig Animalia reference m-DAG for purine metabolism. A file with the Animalia kingdom reference m-DAG for the purine metabolism pathway.
(PDF)

S10 Fig Plantae reference m-DAG for purine metabolism. A file with the Plantae kingdom reference m-DAG for the purine metabolism pathway.
(PDF)

S11 Fig Fungi reference m-DAG for purine metabolism. A file with the Fungi kingdom reference m-DAG for the purine metabolism pathway.
(PDF)

S12 Fig Protista reference m-DAG for purine metabolism. A file with the Protista kingdom reference m-DAG for the purine metabolism pathway.
(PDF)

S13 Fig Bacteria reference m-DAG for purine metabolism. A file with the Bacteria kingdom reference m-DAG for the purine metabolism pathway.
(PDF)

S14 Fig Archaea reference m-DAG for purine metabolism. A file with the Archaea kingdom reference m-DAG for the purine metabolism pathway.
(PDF)

S15 Fig Glycolysis m-DAG dendrogram. A dendrogram obtained with the hierarchical clustering of the m-DAGs for the glycolysis pathway using the distance defined in this paper.
(PDF)

S16 Fig Purine m-DAG dendrogram. A dendrogram obtained with the hierarchical clustering of the m-DAGs for the purine metabolism pathway using the distance defined in this paper.
(PDF)

## References

[1] Sweetlove LJ, Fell D, Fernie AR. Getting to grips with the plant metabolic network. *Biochem J*, 409:27–41, 2008 10.1042/BJ20071115 18062772

[2] Rison SC, Thornton JM. Pathway evolution, structurally speaking. *Current Opinion in Structural Biology*, 12:374–382, 2002 10.1016/S0959-440X(02)00331-7 12127458

[3] Ebenhöh O, Handorf T, Heinrich R. Structural analysis of expanding networks. *Genome Information*, 15:35–45, 2004. 15712108

[4] Steinway SN, Biggs MB, Loughran TP Jr, Papin JA, Albert R. Inference of network dynamics and metabolic interactions in the gut microbiome. *PLoS Comput Biol*, 11(6):e1004338, 2015 10.1371/journal.pcbi.1004338 26102287 PMC4478025

[5] Fani R, Fondi M. Origin and evolution of metabolic pathways. *Physics of Life Reviews*, 6:23–52, 2009 10.1016/j.plrev.2008.12.003 20416849

[6] Lazcano A, Miller SL. The origin and early evolution of life: prebiotic chemistry, the pre-rna world and time. *Journal of Molecular Evolution*, 85:793–798, 1996. 10.1016/s0092-8674(00)81263-5 8681375

[7] Lingam M. Interstellar travel and galactic colonization: Insights from percolation theory and the yule process. *Astrobiology*, 16(6):418–426, 2016 10.1089/ast.2015.1411 27213220

[8] Wiechert W. 13c metabolic flux analysis. *Metabolic Engineering*, 3:195–206, 2001 10.1006/mben.2001.0187 11461141

[9] Kauffman KJ, Prakash P, Edwards JS. Advances in flux balance analysis. *Current Opinion in Biotechnology*, 14(5):491—496, 2003 10.1016/j.copbio.2003.08.001 14580578

[10] Price ND, Reed JL, Palsson BØ. Genome-scale models of microbial cells: evaluating the consequences of constraints. *Nature Reviews Microbiology*, 2(11):886–897, 2004. 15494745 10.1038/nrmicro1023

[11] Abaka G, Bıyıkoğlu T, Erten C. Campways: constrained alignment framework for the comparative analysis of a pair of metabolic pathways. *Bioinformatics*, 29(13):i145–i153, 2013 10.1093/bioinformatics/btt235 23812978 PMC3694646

[12] Alberich R, Llabrés M, Sánchez D, Simeoni M, Tuduri M. Mp-align: alignment of metabolic pathways. *BMC Systems Biology*, 8(1):1–16, 2014 10.1186/1752-0509-8-58 24886436 PMC4045882

[13] Ay F, Kellis M, Kahveci T. Submap: aligning metabolic pathways with subnetwork mappings. *Journal of computational biology*, 18(3):219–235, 2011 10.1089/cmb.2010.0280 21385030 PMC3123932

[14] Kanehisa M, Goto S. Kegg: kyoto encyclopedia of genes and genomes. *Nucleic acids research*, 28(1):27–30, 2000 10.1093/nar/28.1.27 10592173 PMC102409

[15] Biomodels Database.

[16] MetaCyc Encyclopedia of Metabolic Pathways.

[17] Tomar N, De RK. Comparing methods for metabolic network analysis and an application to metabolic engineering. *Gene*, 521(1):1–14, 2013 10.1016/j.gene.2013.03.017 23537990

[18] Klamt S. Stelling J. Combinatorial complexity of pathway analysis in metabolic networks. *Molecular biology reports*, 29(1-2):233–236, 2002 10.1023/A:1020390132244 12241063

[19] Trinh CT, Wlaschin A, Srienc F. Elementary mode analysis: a useful metabolic pathway analysis tool for characterizing cellular metabolism. *Applied microbiology and biotechnology*, 81(5):813–826, 2009 10.1007/s00253-008-1770-1 19015845 PMC2909134

[20] Price ND, Reed JL, Papin JA, Wiback SJ, Palsson BØ. Network-based analysis of metabolic regulation in the human red blood cell. *Journal of Theoretical Biology*, 225(2):185–194, 2003 10.1016/S0022-5193(03)00237-6 14575652

[21] KEGG pathway database—Kyoto University Bioinformatics Centre.

[22] Chartrand G, Lesniak L, Zhang P. *Graphs & Digraphs*, *Fifth Edition*. Chapman & Hall/CRC, 5th edition, 2010.

[23] Tarjan RE. Depth–first search and linear graph algorithms. *SIAM journal on computing*, 1(2):146–160, 1972 10.1137/0201010

[24] Woese CR, Fox GE. Phylogenetic structure of the prokaryotic domain: the primary kingdoms. *Proceedings of the National Academy of Sciences*, 74(11):5088–5090, 1977 10.1073/pnas.74.11.5088 PMC432104 270744

[25] Ruggiero MA, Gordon DP, Orrell TM, Bailly N, Bourgoin T, Brusca RC, Cavalier-Smith T, Guiry MD, Kirk PM. A higher level classification of all living organisms. *PloS one*, 10(4):e0119248, 2015 10.1371/journal.pone.0119248 25923521 PMC4418965

[26] Garrity G, Brenner DJ, Krieg NR, Staley JR. *Bergey’s Manual^®^ of Systematic Bacteriology: Volume 2: The Proteobacteria*, *Part B: The Gammaproteobacteria* Bergey’s Manual of Systematic Bacteriology. Springer US, 2007.

[27] Ver Eecke HC, Kelley DS, Holden JF. Abundances of hyperthermophilic autotrophic fe(iii) oxide reducers and heterotrophs in hydrothermal sulfide chimneys of the northeastern pacific ocean. *Appl Environ Microbiol*, 75(1):242–245, 2009 10.1128/AEM.01462-08 18978076 PMC2612199

[28] Webb EC. *Enzyme nomenclature 1992: recommendations of the Nomenclature Committee of the International Union of Biochemistry and Molecular Biology on the nomenclature and classification of enzymes*. San Diego: Published for the International Union of Biochemistry and Molecular Biology by Academic Press, 1992.

[29] Hattori M, Okuno Y, Goto S, Kanehisa M. Development of a chemical structure comparison method for integrated analysis of chemical and genomic information in metabolic pathways. *Journal of American Chemical Society*, 125:11853–11865, 2003 10.1021/ja036030u 14505407

