# Correction: Metabolomics analysis: Finding out metabolic building blocks

**DOI:** 10.1371/journal.pone.0186626

**Published:** 2017-10-12

**Authors:** Ricardo Alberich, José A. Castro, Mercè Llabrés, Pere Palmer-Rodríguez

There is information missing from the Funding section. The correct funding information is as follows: The research reported in this paper has been partially supported by the Spanish Government, through project DPI2015-67082-P (AEI/FEDER, UE), and the “Programa Pont La Caixa per a grups de recerca de la UIB”. The funders had no role in study design, data collection and analysis, decision to publish, or preparation of the manuscript.
