# Supplementary figures and images for: Metabolomics analysis: Finding out metabolic building blocks
Source: PLoS One. 2017 May 11;12(5):e0177031. doi: 10.1371/journal.pone.0177031 (PMC5426688; doi:10.1371/journal.pone.0177031)

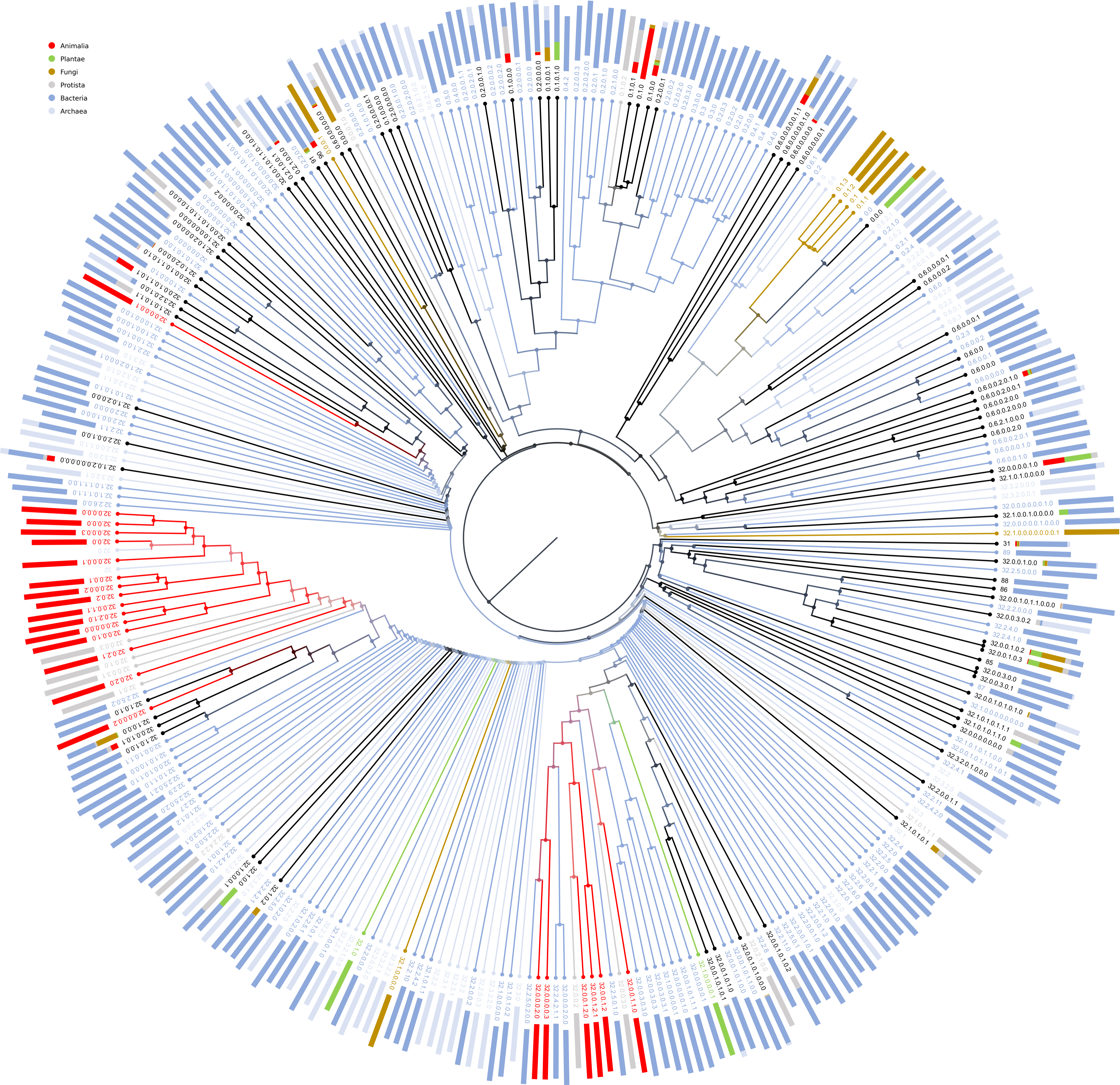

Supplement: S1 Fig — A dendrogram obtained with the hierarchical clustering of the MBBs using the distances in S3 Table. (PDF) [file pone.0177031.s015.pdf]

Animalia  
Plantae  
Fungi  
Protista  
Bacteria  
Archaea

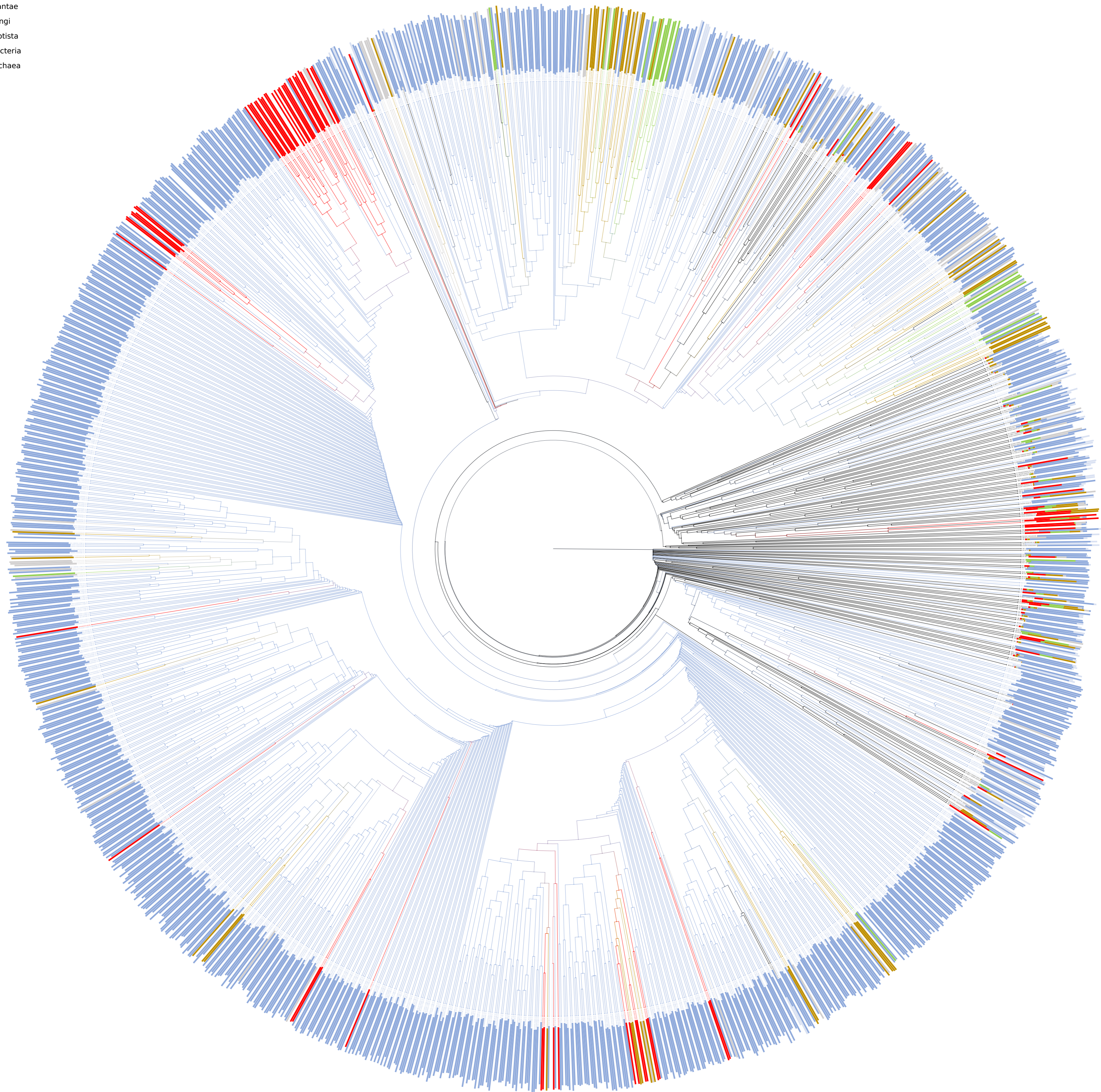

Supplement: S2 Fig — A dendrogram obtained with the hierarchical clustering of the MBBs using the distances in S4 Table. (PDF) [file pone.0177031.s016.pdf]

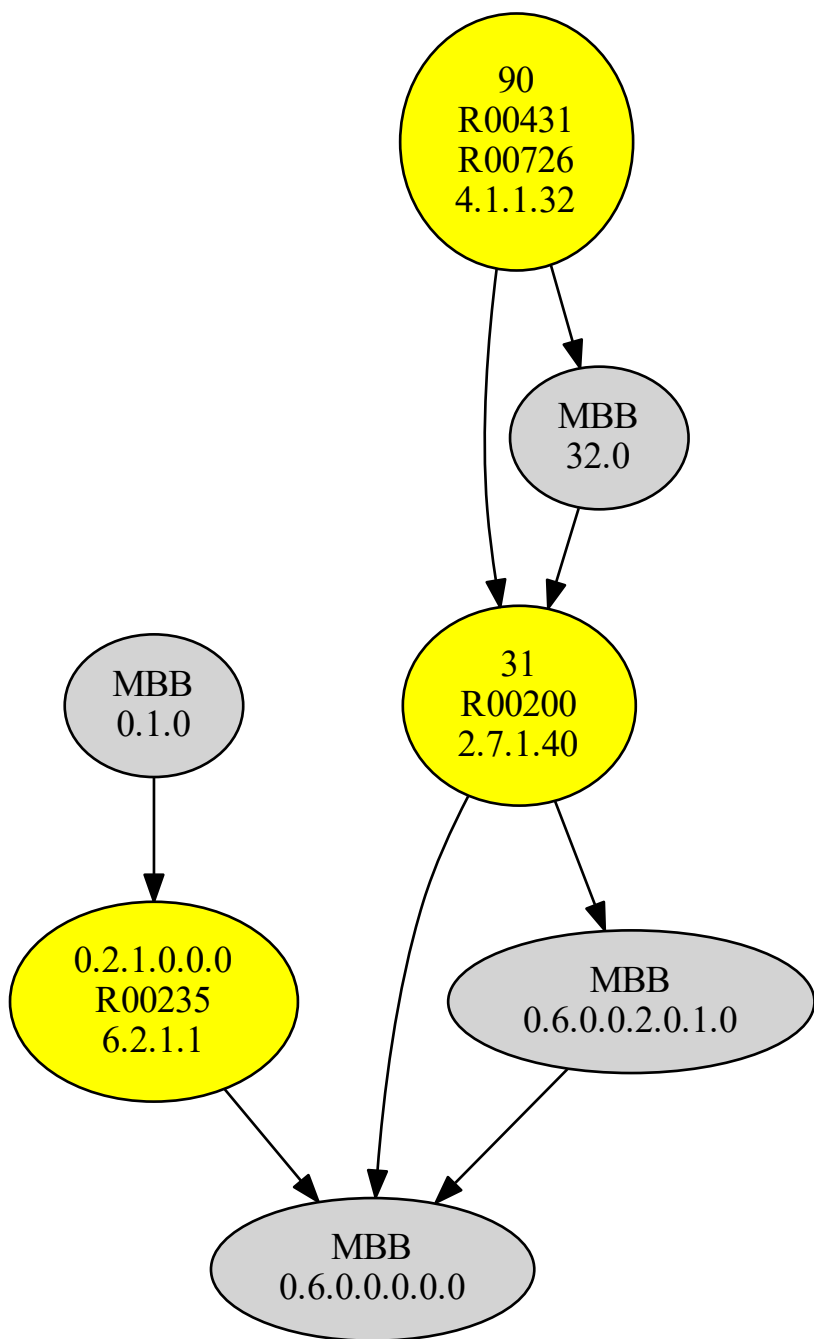

Supplement: S3 Fig — A file with the Animalia kingdom reference m-DAG for the glycolysis pathway. (PDF) [file pone.0177031.s017.pdf]

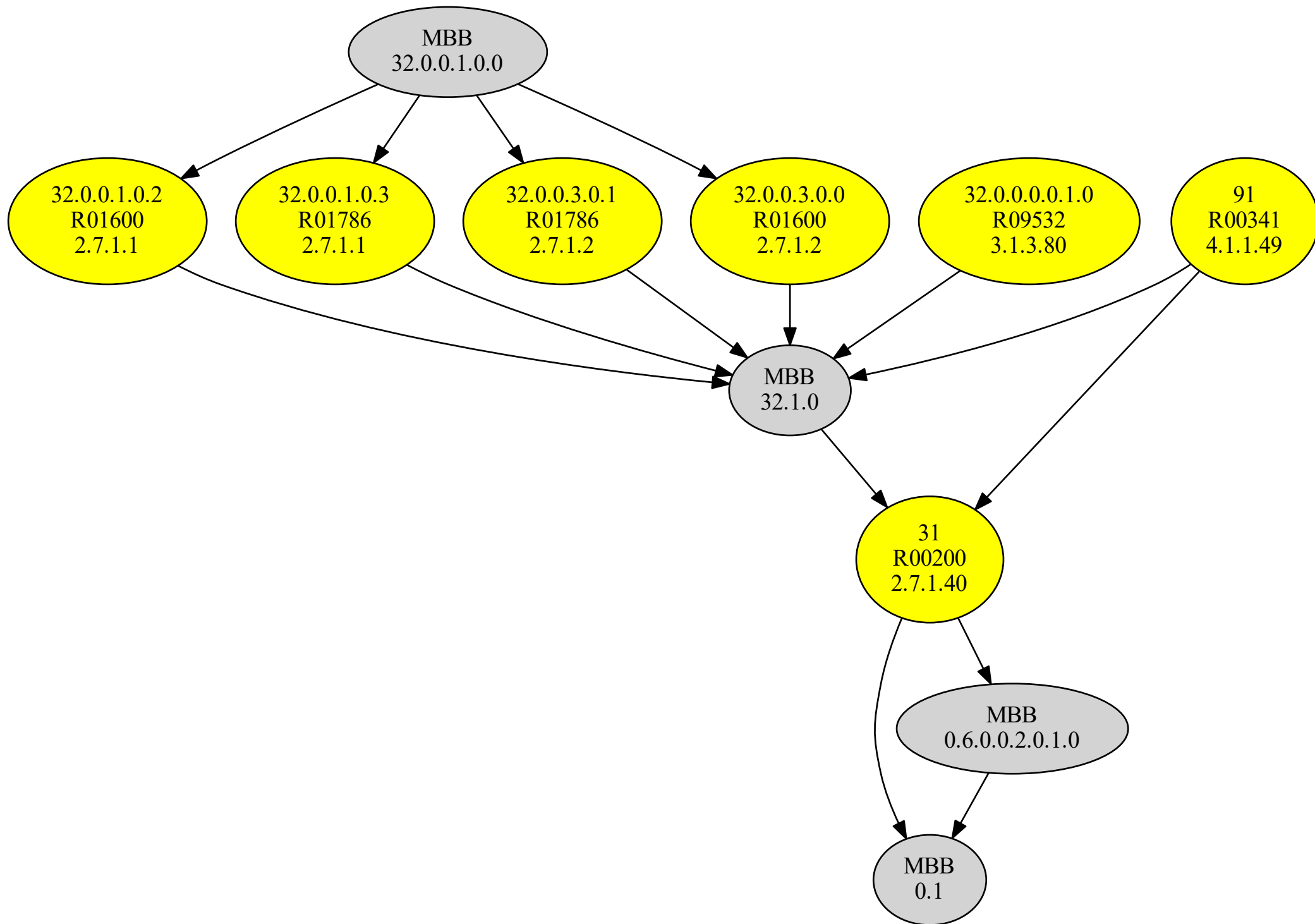

Supplement: S4 Fig — A file with the Plantae kingdom reference m-DAG for the glycolysis pathway. (PDF) [file pone.0177031.s018.pdf]

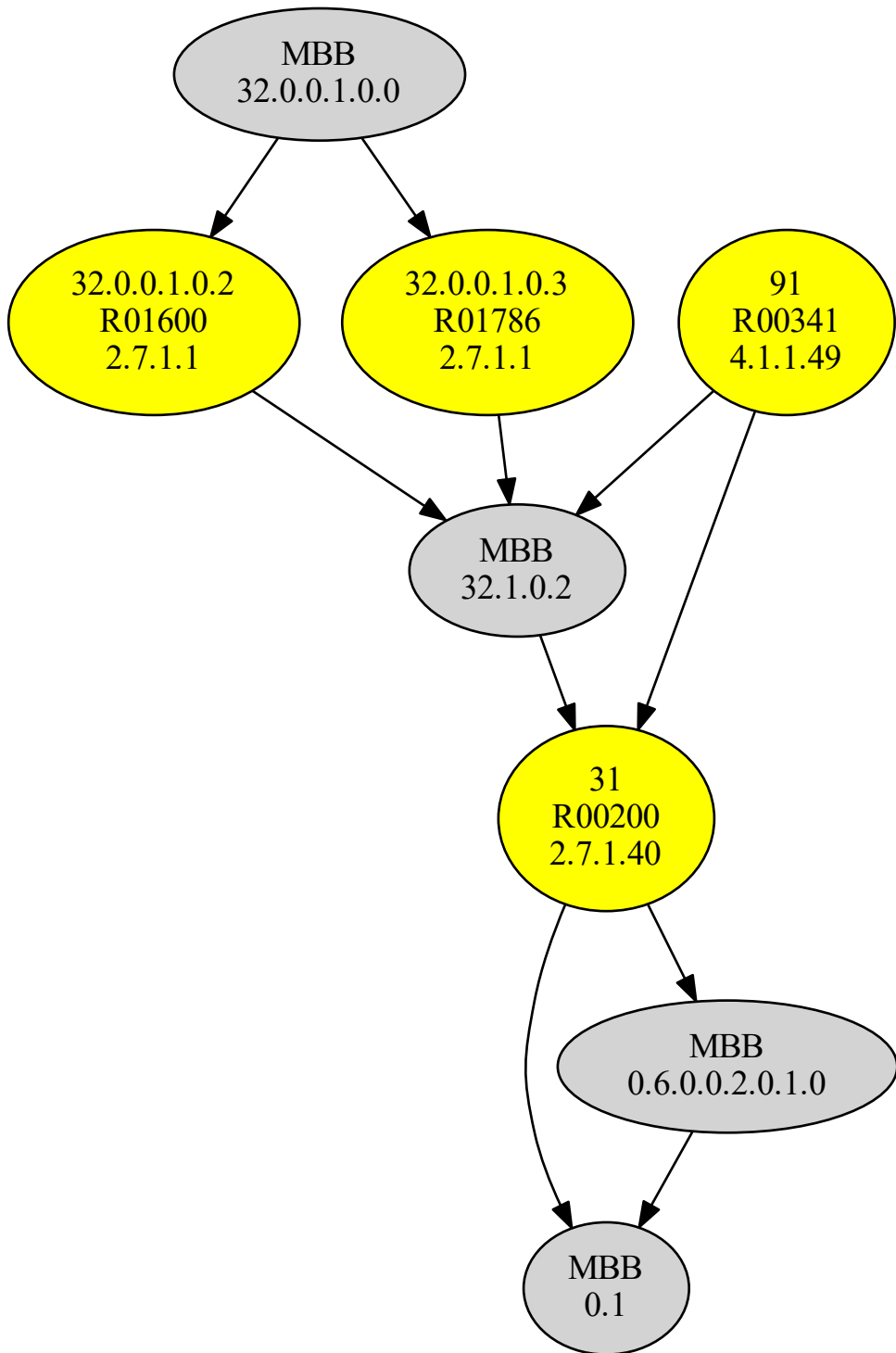

Supplement: S5 Fig — A file with the Fungi kingdom reference m-DAG for the glycolysis pathway. (PDF) [file pone.0177031.s019.pdf]

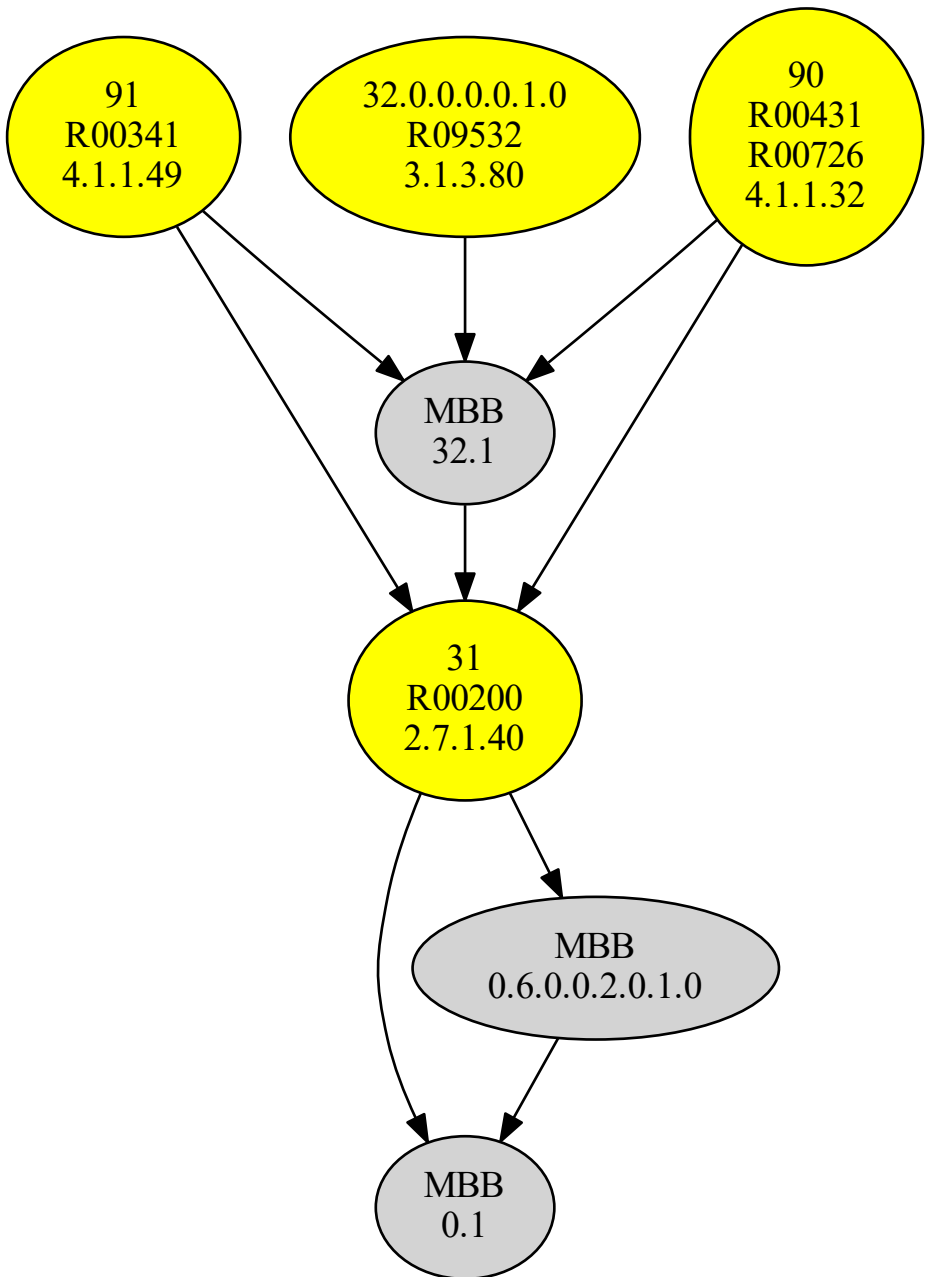

Supplement: S6 Fig — A file with the Protista kingdom reference m-DAG for the glycolysis pathway. (PDF) [file pone.0177031.s020.pdf]

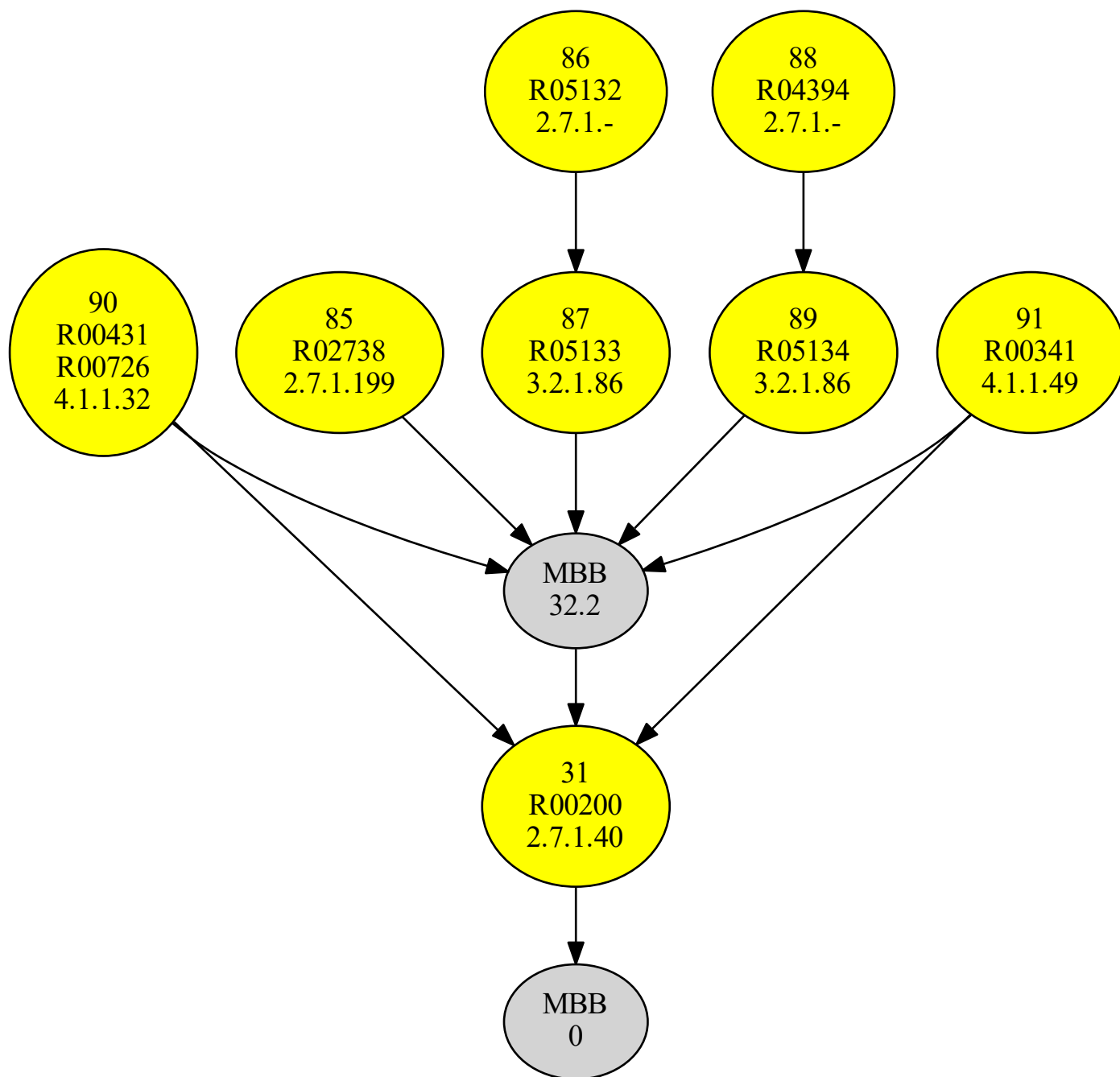

Supplement: S7 Fig — A file with the Bacteria kingdom reference m-DAG for the glycolysis pathway. (PDF) [file pone.0177031.s021.pdf]

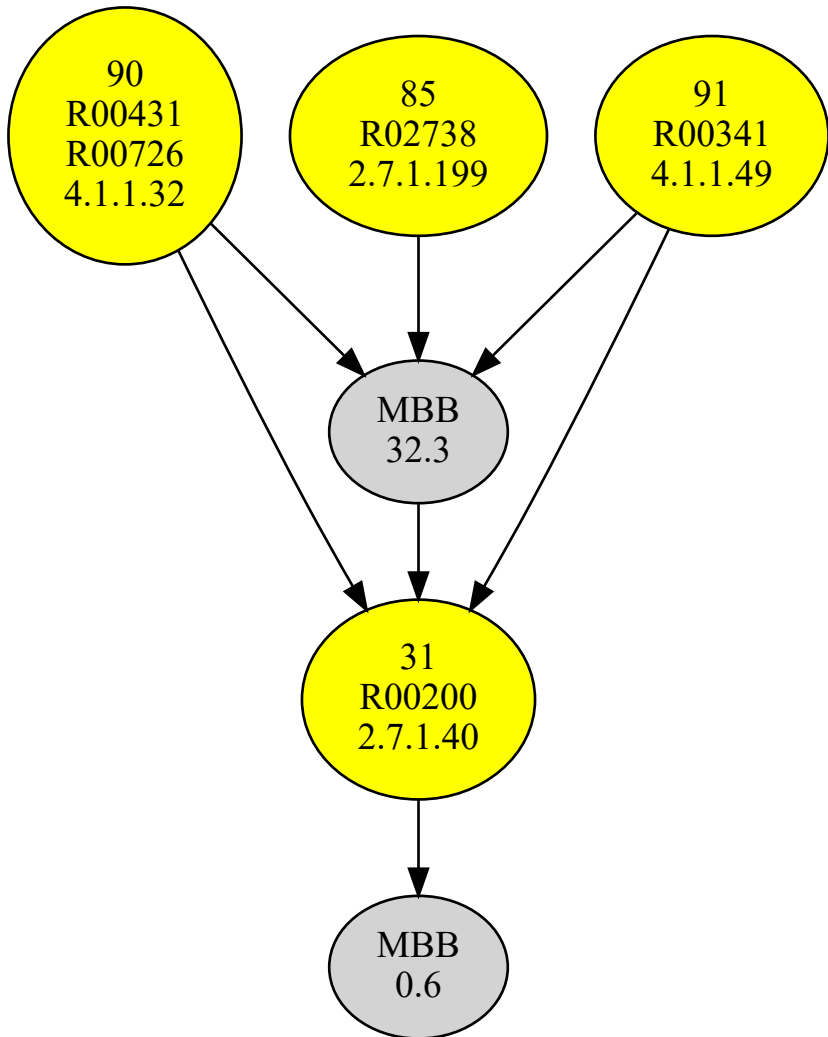

Supplement: S8 Fig — A file with the Archaea kingdom reference m-DAG for the glycolysis pathway. (PDF) [file pone.0177031.s022.pdf]

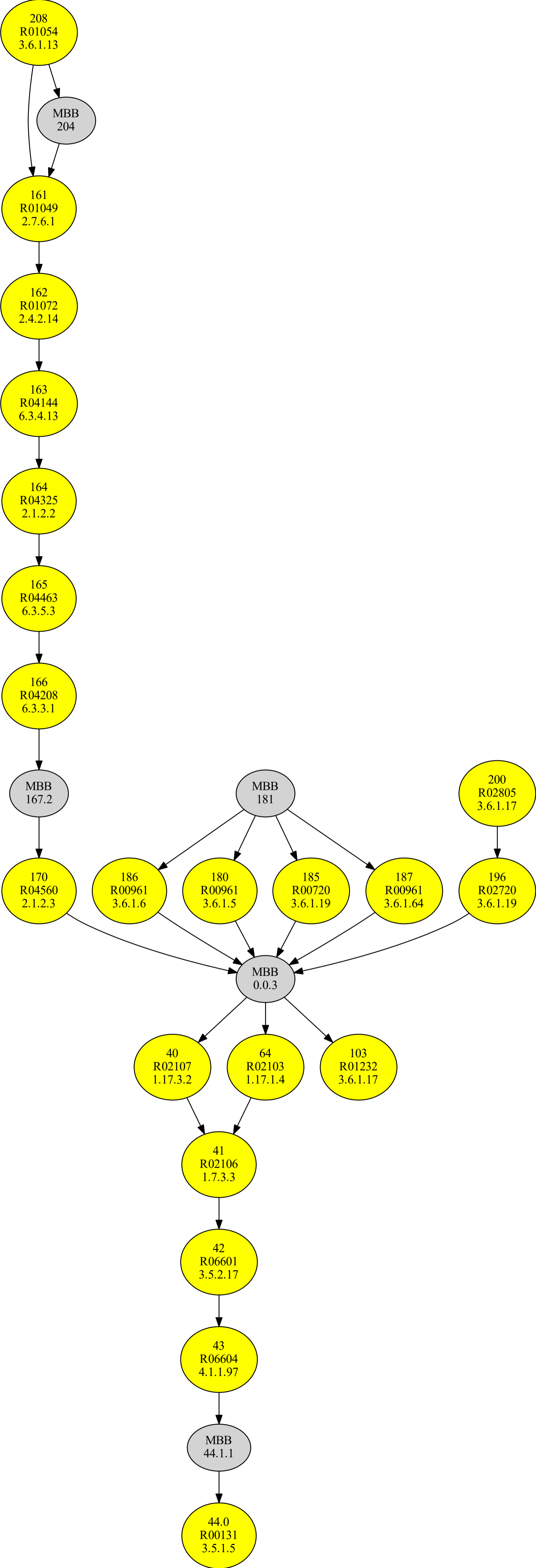

Supplement: S9 Fig — A file with the Animalia kingdom reference m-DAG for the purine metabolism pathway. (PDF) [file pone.0177031.s023.pdf]

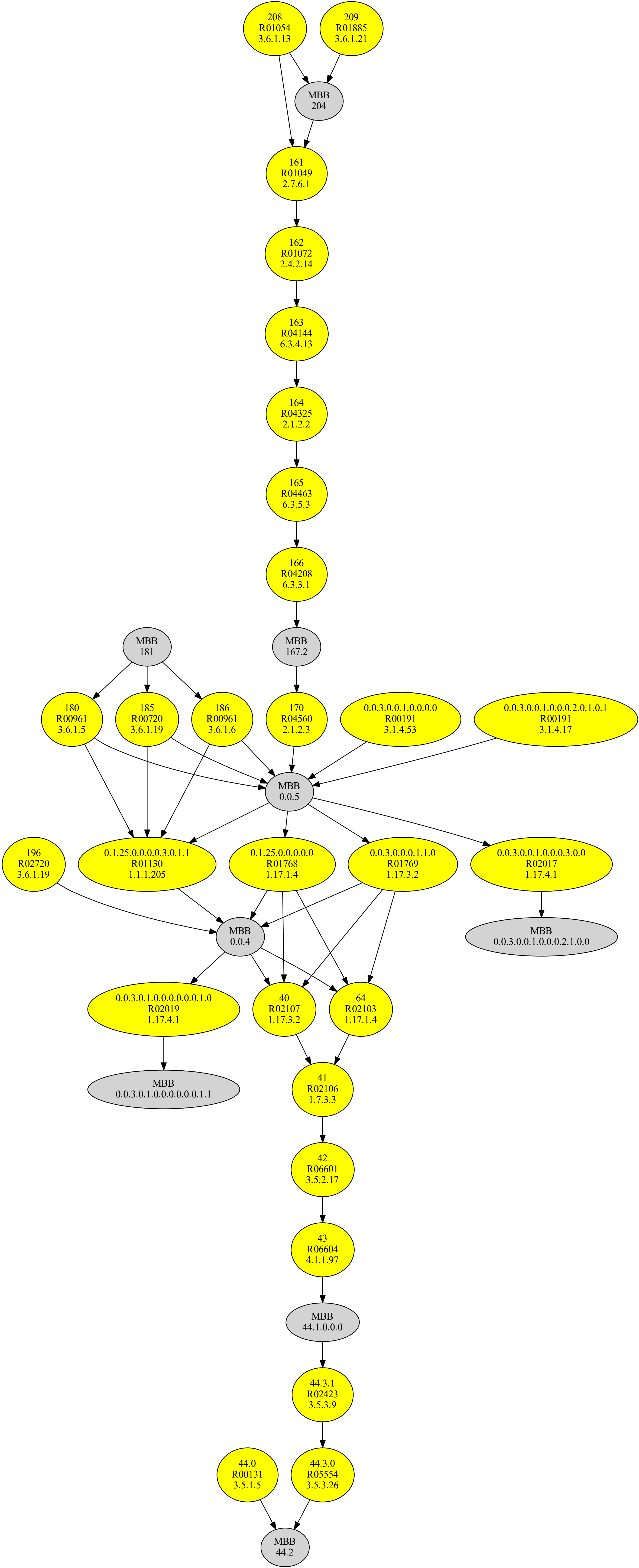

Supplement: S10 Fig — A file with the Plantae kingdom reference m-DAG for the purine metabolism pathway. (PDF) [file pone.0177031.s024.pdf]

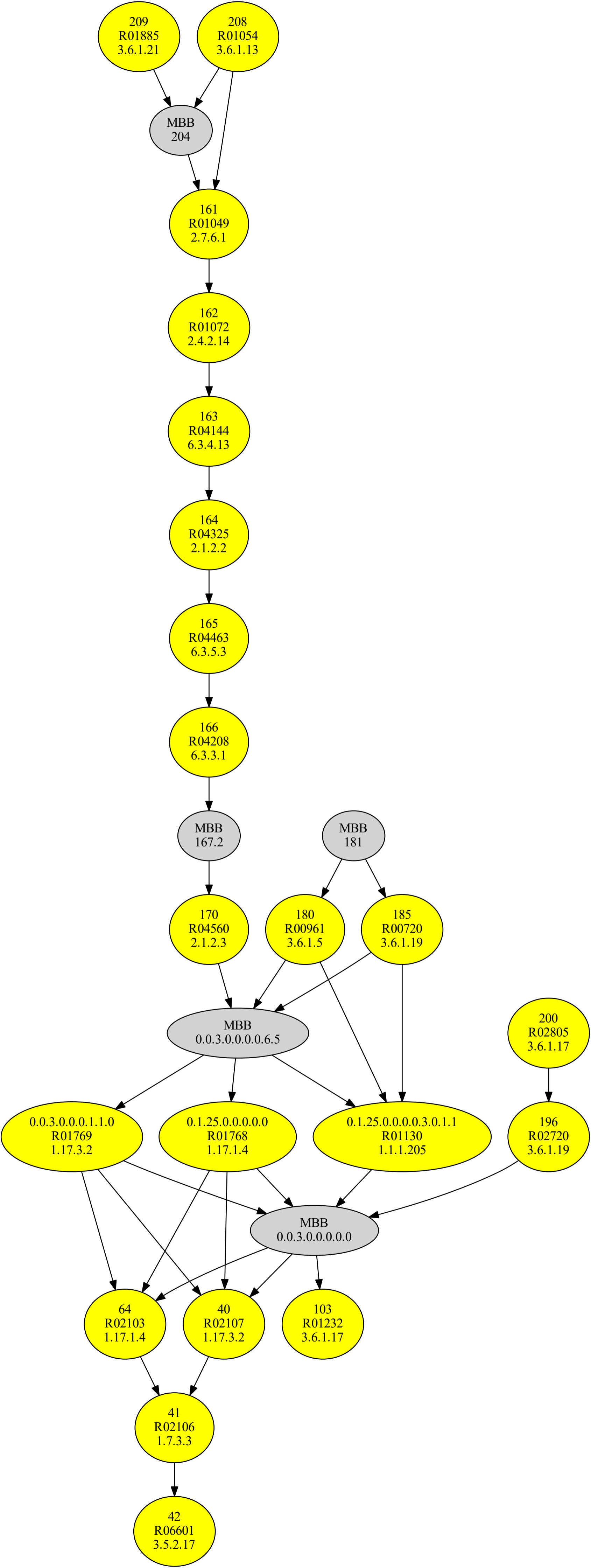

Supplement: S11 Fig — A file with the Fungi kingdom reference m-DAG for the purine metabolism pathway. (PDF) [file pone.0177031.s025.pdf]

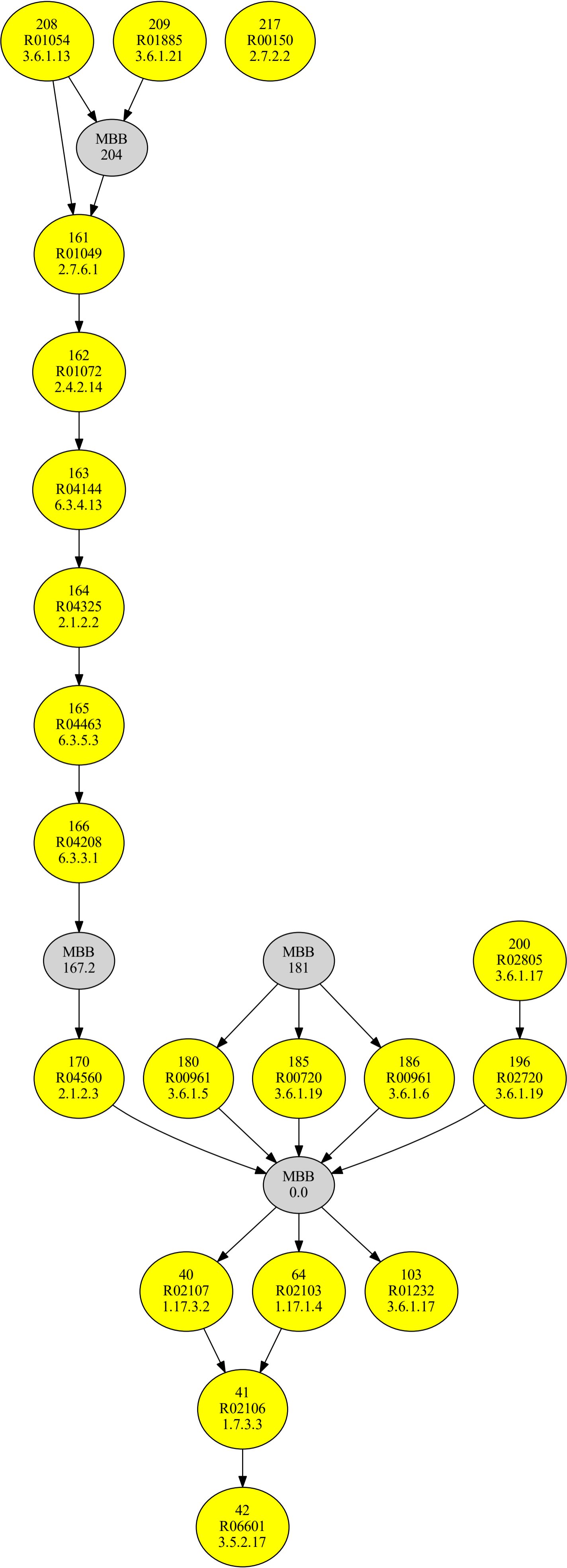

Supplement: S12 Fig — A file with the Protista kingdom reference m-DAG for the purine metabolism pathway. (PDF) [file pone.0177031.s026.pdf]

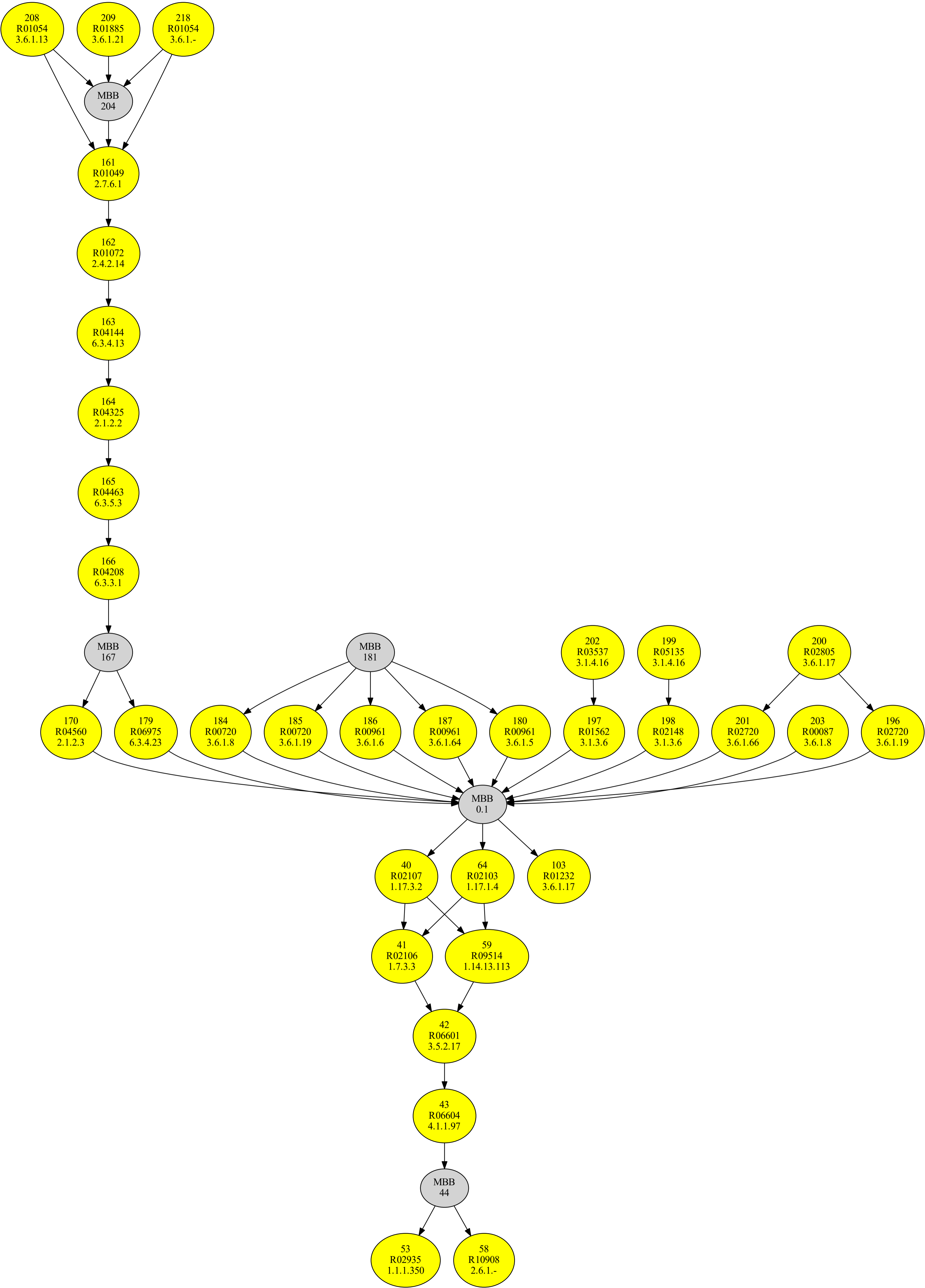

Supplement: S13 Fig — A file with the Bacteria kingdom reference m-DAG for the purine metabolism pathway. (PDF) [file pone.0177031.s027.pdf]

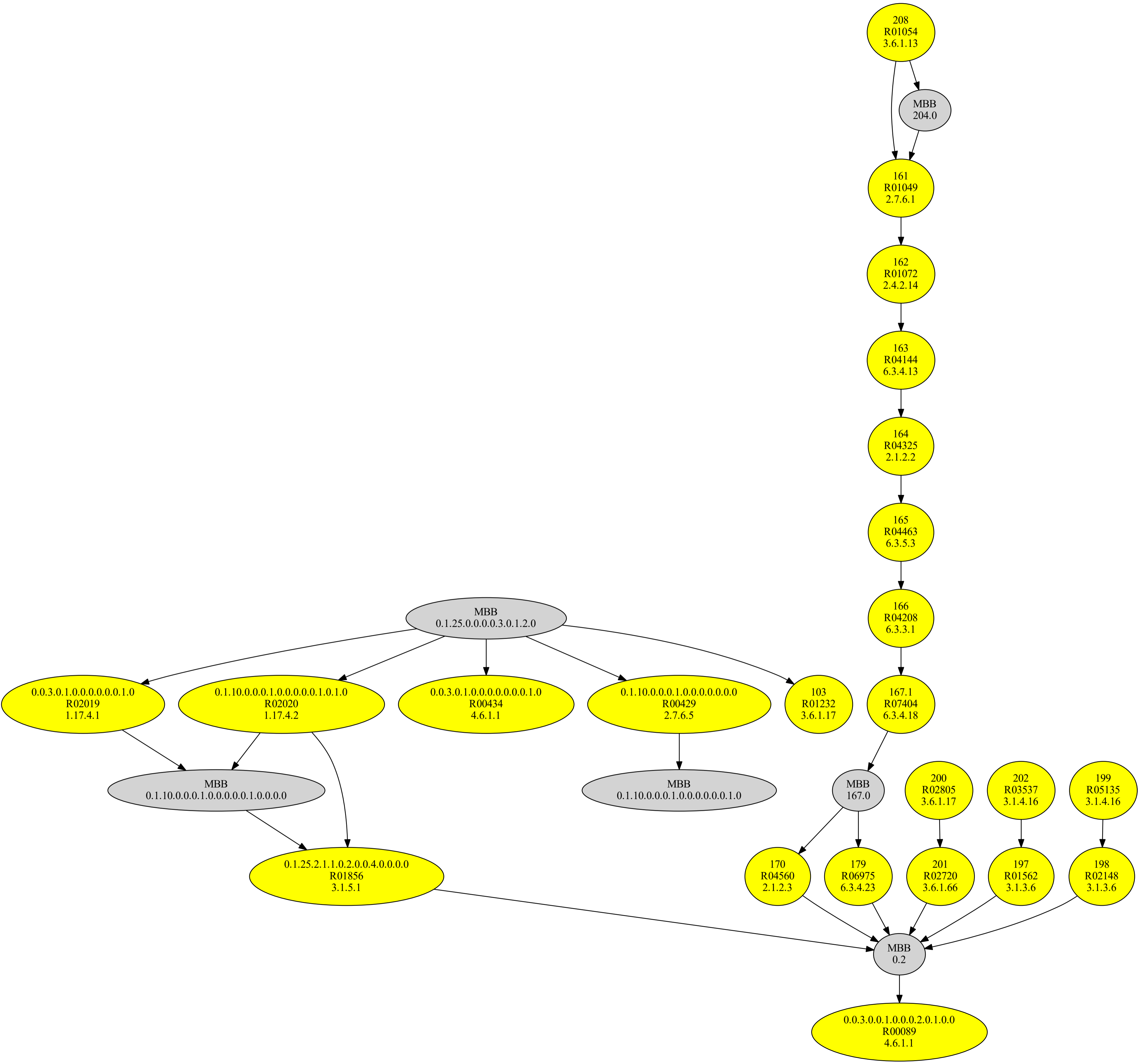

Supplement: S14 Fig — A file with the Archaea kingdom reference m-DAG for the purine metabolism pathway. (PDF) [file pone.0177031.s028.pdf]

- Animalia
- Plantae
- Fungi
- Protista
- Bacteria
- Archaea

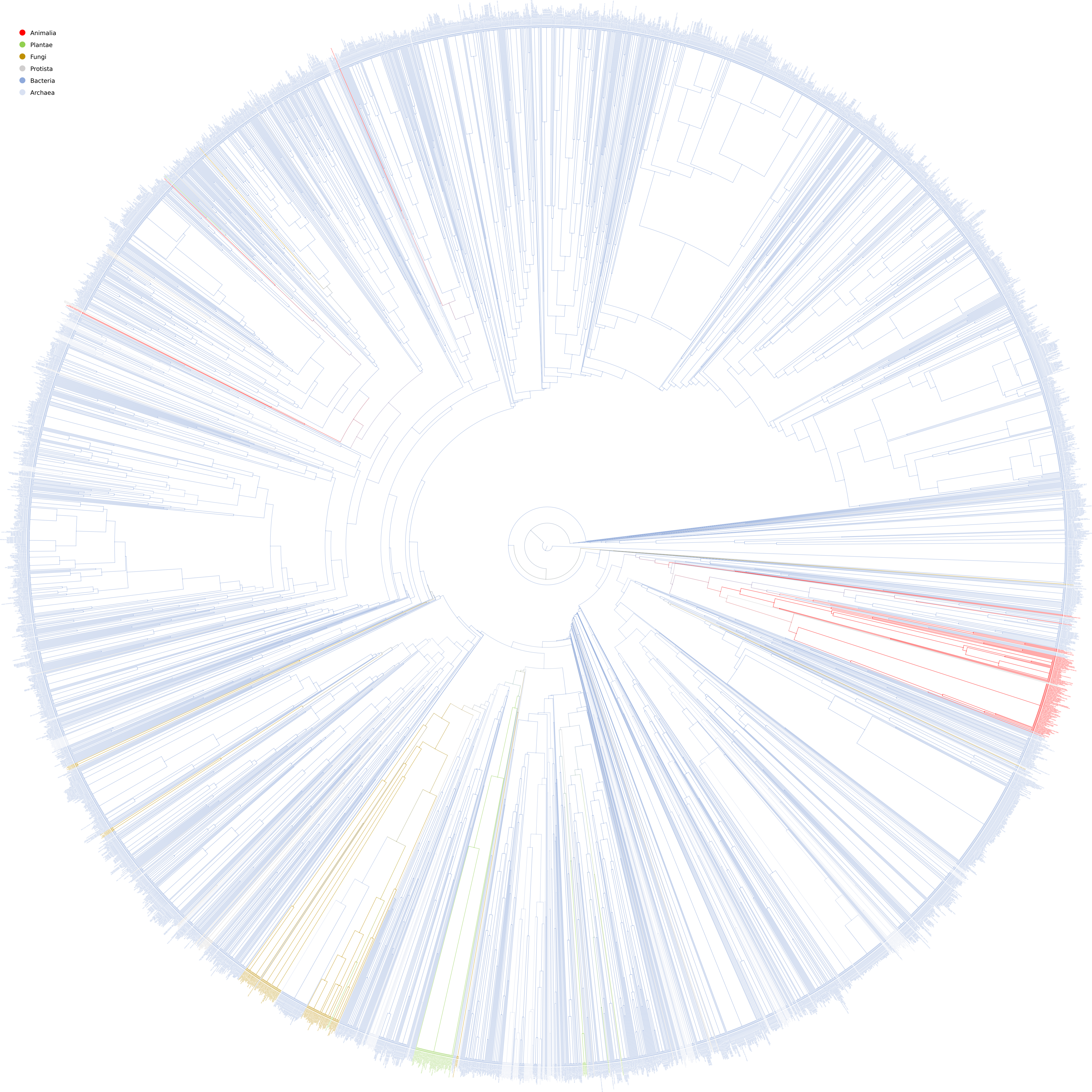

Supplement: S15 Fig — A dendrogram obtained with the hierarchical clustering of the m-DAGs for the glycolysis pathway using the distance defined in this paper. (PDF) [file pone.0177031.s029.pdf]

- Animalia
- Plantae
- Fungi
- Protista
- Bacteria
- Archaea

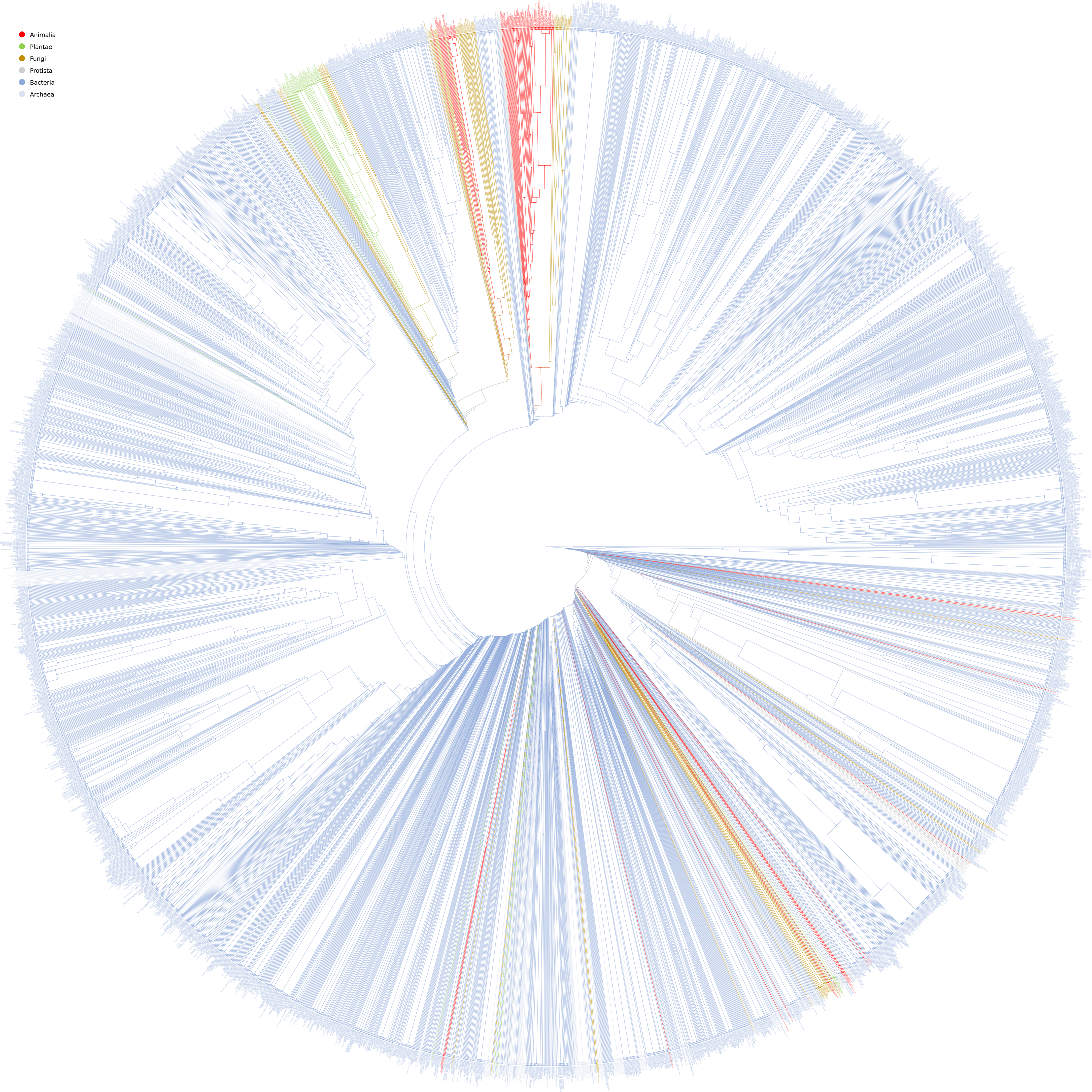

Supplement: S16 Fig — A dendrogram obtained with the hierarchical clustering of the m-DAGs for the purine metabolism pathway using the distance defined in this paper. (PDF) [file pone.0177031.s030.pdf]
